# Hypoxia-mediated SUMOylation of FADD exacerbates endothelial cell injury via the RIPK1-RIPK3-MLKL signaling axis

**DOI:** 10.1038/s41419-025-07441-2

**Published:** 2025-02-21

**Authors:** Liming Yang, Yilin Wen, Zhiyi Yuan, Dezhang Zhao, Ping Weng, Yueyue Li, Qingyang Chen, Wanping Zhang, Hui Hu, Chao Yu

**Affiliations:** 1 College of Pharmacy, Chongqing Medical University, Chongqing, China; 2 Chongqing Key Laboratory for Pharmaceutical Metabolism Research, Chongqing, China

**Keywords:** Sumoylation, Necroptosis, Sumoylation

## Abstract

Vascular endothelial cells are the predominant cell type in the cardiovascular system, and their dysfunction and death following hypoxic injury contribute to vascular lesions, playing an essential role in cardiovascular disease. Despite its importance, the mechanisms underlying vascular endothelial cell injury under hypoxia and potential therapeutic interventions remain poorly understood. Here, we constructed both an in vivo hypoxia model in C57BL/6 mice and an in vitro hypoxia model in HUVEC cells. Our findings demonstrated that hypoxia induces necroptosis in vascular endothelial cells and exacerbates inflammatory injury in vivo and in vitro, as evidenced by immunofluorescence and western blot. We identified FADD as a critical regulator of hypoxia-mediated necroptosis, with FADD knockdown significantly reversing hypoxia-induced necroptosis. Mechanistically, hypoxia affected protein conformation through SUMOylation of FADD and competitively inhibited its ubiquitination, leading to an increase in protein half-life and protein level of FADD. Furthermore, SUMOylation increased the interaction between FADD and RIPK1 and induced the formation of the FADD-RIPK1-RIPK3 complex, thereby promoting necroptosis in vascular endothelial cells. The SUMOylation inhibitor ginkgolic acid (GA) notably reduced hypoxia-induced vascular endothelial injury and inflammatory responses in male mice. Taken together, our research has uncovered a new process by which SUMOylation of FADD regulates hypoxia-induced necroptosis in endothelial cells, providing potential therapeutic targets for hypoxia-related cardiovascular diseases.

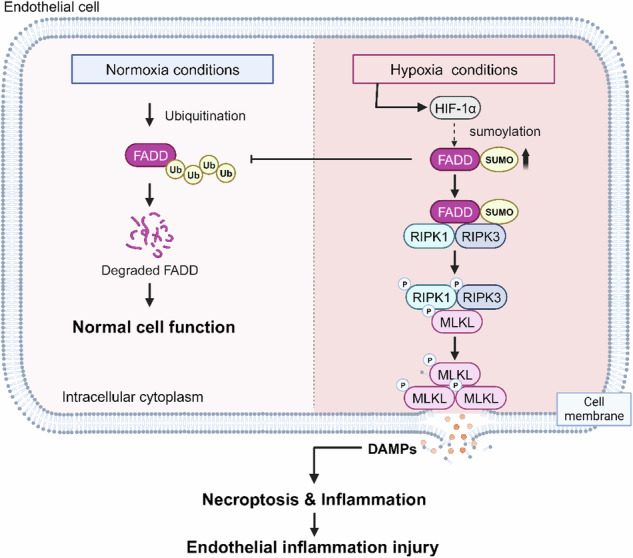

## Introduction

Hypoxia is a disorder caused by a reduction in the availability of oxygen to tissues or organs. It is associated with a variety of human diseases, including ischemic damage to the brain, heart, liver, and kidney [1–3]. Under hypoxia, HIF-1α accumulates in cells to promote cell adaptation and survival [4]. However, under extensive severe hypoxic stress, cells activate death mechanisms leading to cell death. Vascular endothelial cell (ECs) is the most common cell type in the cardiovascular system, being the first layer to be exposed to changes in oxygen levels [1]. Hypoxia and HIF signaling stimulate endothelial cell activation, leading to endothelial dysfunction [5]. Dysfunction of ECs ultimately leads directly not only to altered vasoconstrictor activity, intimal thickening, and remodeling but also to the recruitment of monocytes, which induces an increase in inflammatory cytokines, leading to further vascular lesions [6–8]. However, the molecular regulatory mechanism of how hypoxia mediates vascular endothelial cell injury remains elucidated.

Necroptosis, a novel type of programmed cell death, involves cell swelling, plasma membrane rupture, loss of cytoplasmic contents, cell permeabilization, and release of damage-associated molecular patterns (DAMP) [9, 10]. Hypoxia is closely associated with necroptosis. By inhibiting necroptosis, it is possible to improve hypoxia-mediated retinal neoangiogenesis [11] and facilitate hypoxic brain injury as well as ischemic brain injury caused by middle cerebral artery obstruction [12, 13]. However, how necroptosis is involved in the pathogenesis of ischemic-hypoxic vascular endothelial injury has yet to be studied.

FADD (Fas-associated via death domain) is a crucial junction protein in death receptor-mediated apoptosis [14]. In addition to its role in apoptosis, FADD is involved in other non-apoptotic processes, such as necroptosis. Caspase 8, dependent on FADD activation, inhibits necroptosis by shearing RIPK1/RIPK3 [15]. Interestingly, FADD can promote necroptosis in some studies, and the FADD-RIPK1-RIPH3-NEMO complex can induce BAX/BAK-dependent mitochondrial bioenergetic catabolism to promote TNFα-driven necroptosis [16, 17]. The precise role of FADD remains elusive and opposite. Thus, the role and molecular mechanism of FADD in hypoxia-induced necroptosis need to be further investigated.

SUMOylation is one of the post-transcriptional modifications (PTMs) in eukaryotic protein. SUMOylation and ubiquitination are similar but functionally distinct. Ubiquitin-modified proteins mainly make them recognized and degraded by the proteasome. In contrast, SUMO-modified proteins are more stable, while SUMOylation can modulate protein-protein interactions to mediate target protein localization and functional regulation [18, 19]. Studies have shown that FADD has three SUMOylation sites: K120, K125, and K149 [20, 21]. Here, we identified a novel mechanism of SUMOylation of FADD in necroptosis and its impact on hypoxic endothelial injury. Under hypoxia, SUMOylation of FADD competitively inhibited its ubiquitination by affecting the protein conformation, leading to an increase in the protein half-life and protein level of FADD while inducing the formation of the FADD-RIPK1-RIPK3 complex, which promotes necroptosis in vascular ECs. Our findings provide new insights into hypoxia-mediated necroptosis, suggesting that targeting SUMOylation and FADD has the potential to prevent and treat vascular ischemic injury and related cardiovascular diseases.

## Result

### Hypoxia mediates vascular endothelial damage with concomitant inflammatory responses

**Fig. 1:**
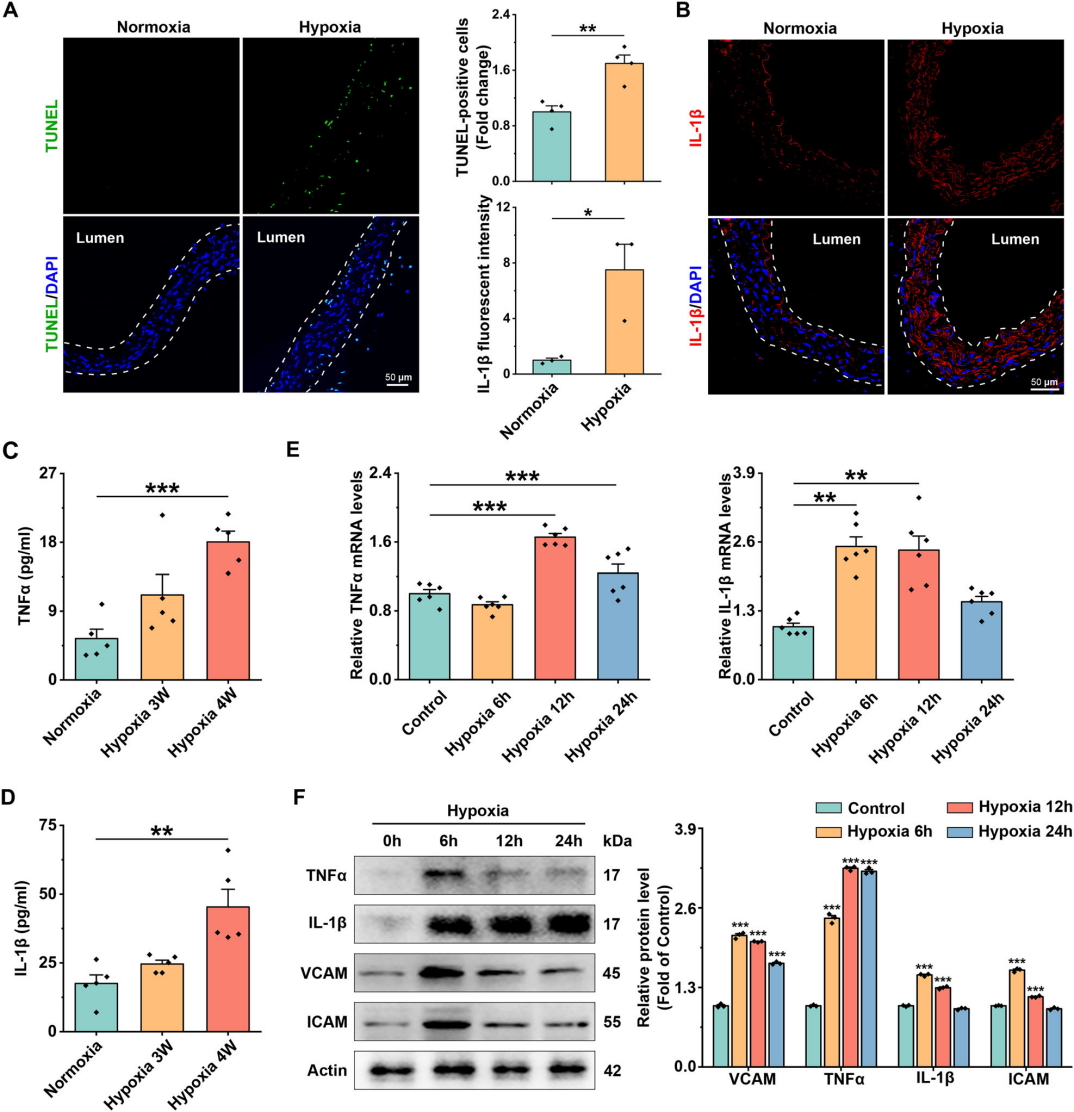
Hypoxia mediates vascular endothelial damage with concomitant inflammatory responses. **A** Representative images of TUNEL (green) staining of mouse aortic vessel sections after 4 weeks of hypoxia and results of statistical analysis. Scale bar = 50 μm, *n* = 4. **B** Representative images of IL-1β (red) staining of mouse aortic vessels after 4 weeks of hypoxia treatment and results of statistical analysis. Scale bar = 50 μm, *n* = 3. **C** Serum levels of the inflammatory cytokine TNFα were determined by Elisa assay, *n* = 5. **D** Serum levels of the inflammatory cytokine IL-1β were determined by Elisa assay, *n* = 5. **E** Changes in IL-1β mRNA and TNFα mRNA expression levels in hypoxia-treated HUVEC cells at different periods, with Actin as an internal reference gene, *n* = 6. **F** Representative immunoblot bands and statistical analyses of IL-1β, TNFα, ICAM, and VCAM in hypoxia-treated HUVEC cells at different time intervals, Actin was used as the reference protein, *n* = 3. Data are expressed as mean ± SEM. Relevant experiments in this section were performed independently at least three times. *p < 0.05, **p < 0.01, ***p < 0.001.

To investigate the effects of hypoxia on vascular function, we constructed an in vivo hypoxia model in C57BL/6 mice, which were placed in an animal hypoxia chamber (10% O_2_) for 1, 2, 3, and 4 weeks. We measured dead cells in the aortic vasculature of C57BL/6 mice (8–10 weeks old). The number of TUNEL^+^ cells in the aorta increased progressively starting at 3 weeks (Fig. 1A, Supplementary S1A). Additionally, TUNEL/CD31 fluorescence staining revealed that these TUNEL^+^ cells were primarily localized within the vascular endothelium (Supplementary S1C). An inflammatory response often accompanies vascular injury. We observed that hypoxia for 4 weeks could significantly increase vascular inflammation by IL-1β immunofluorescence (Fig. 1B). The serum levels of IL-1β and TNFα were significantly increased (Fig. 1C, D). Similarly, in the HUVEC with hypoxia intervention, the expression of TNFα reached a peak at hypoxia 12 h, the protein expressions of other inflammatory factors (IL-1β, ICAM, VCAM) reached their peak after 6 h of hypoxia (Fig. 1E, F). These results suggest that hypoxia induces vascular EC dysfunction and cell death.

### Hypoxia induces necroptosis in vascular endothelial cells

**Fig. 2:**
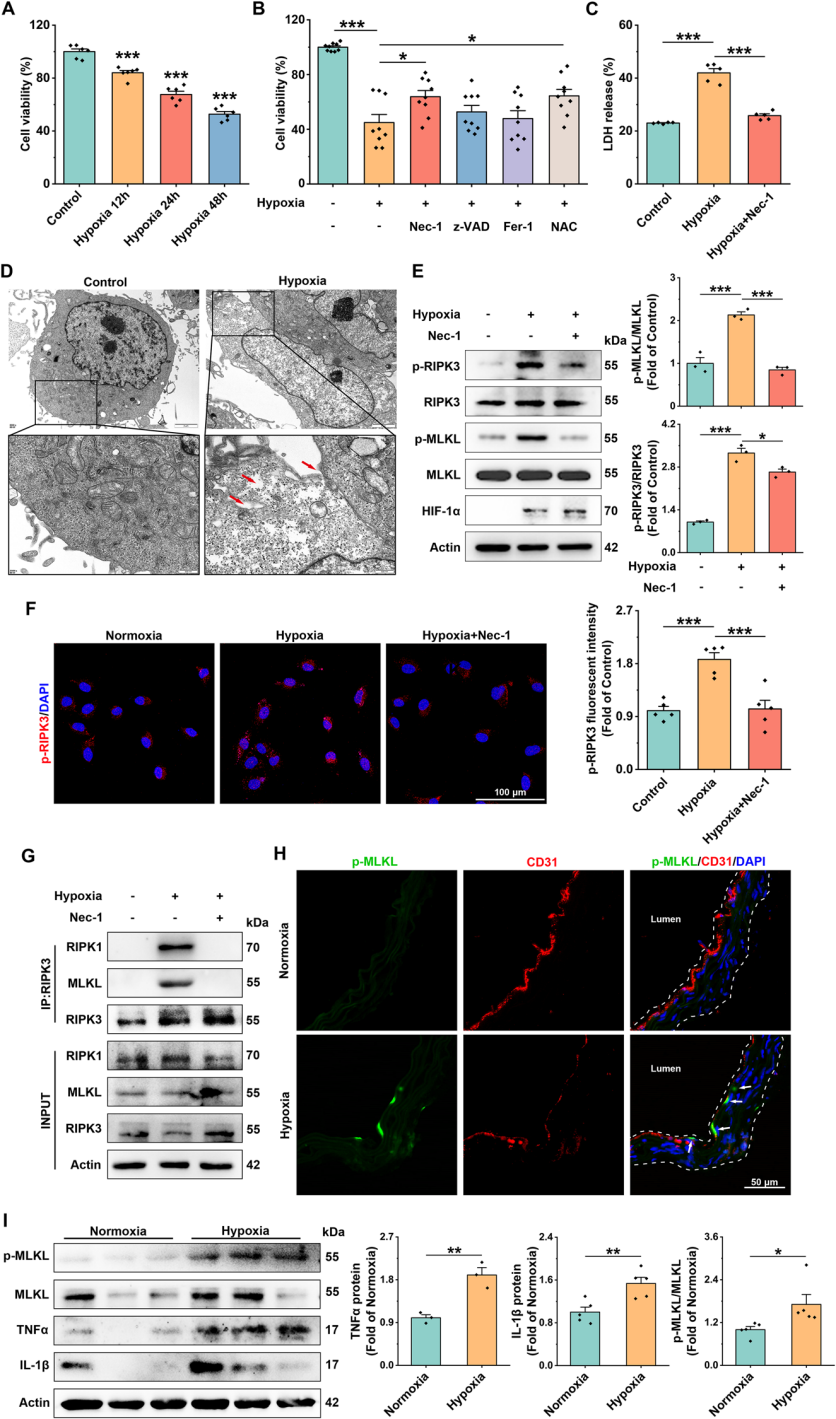
Hypoxia induces necroptosis in vascular endothelial cells. **A** CCK8 assay was performed to determine changes in cell survival after hypoxia, *n* = 6. **B** Changes in HUVEC cell survival after hypoxia treatment in the presence of 50 μM Nec-1, 100 μM z-VAD-fmk, 10 μM Fer-1, and 1 mM NAC, *n* = 9. **C** Changes in cytotoxicity after hypoxia as determined by LDH release, *n* = 5. **D** Representative transmission electron microscopy images of HUVEC after hypoxia treatment. HUVEC in the hypoxia group exhibited translucent cytoplasm, loss of cytoplasmic contents, and membrane damage. Scale bar = 2 μm (merged image) and 500 nm (magnified image). **E** Representative immunoblot bands and statistical analyses of HIF-1α, RIPK3, p-RIPK3, MLKL, p-MLKL in Ctrl, hypoxia, and hypoxia+Nec-1 groups, Actin was used as the reference protein, *n* = 3. **F** Representative confocal images and statistical analysis results of p-RIPK3 (red) after 12 h of hypoxia treatment. scale bar = 100 μm, *n* = 5. **G** HUVEC from normoxic and hypoxic groups were lysed and immunoprecipitated with anti-RIPK3 antibodies, followed by immunoblotting with anti-RIPK3, anti-MLKL, and anti-RIPK1 antibodies. **H** Representative images of CD31 (green) and p-MLKL (red) staining of aortic vascular sections from mice after 4 weeks of hypoxic treatment. Arrows indicate CD31 and p-MLKL co-localized fractions. scale bar = 50 μm. **I** Representative immunoblot bands and statistical analyses of aortic vascular tissues for IL-1β, TNFα, MLKL, and p-MLKL in mice after 4 weeks of hypoxia treatment, Actin was used as the reference protein, *n* = 5. Data are expressed as mean ± SEM. Relevant experiments in this section were performed independently at least three times. *p < 0.05, **p < 0.01, ***p < 0.001.

Since hypoxia increased TUNEL^+^ levels in vascular ECs, we further determined the cell viability of HUVEC by CCK8 assay. The results showed that hypoxia significantly inhibited the cell viability in a time-dependent manner (Fig. 2A). To confirm the cytotoxic effect of hypoxia on HUVEC, the cell viability was examined under various inhibitors, including caspase inhibitor z-VAD-fmk, RIPK1 kinase inhibitor Nec-1, lipid ROS scavenger Fer-1, and antioxidant NAC. The results showed that Nec-1 and NAC partially prevented hypoxia (48 h)-induced cell death (Fig. 2B), and Nec-1 significantly prevented necrotic cells after hypoxia (48 h) treatment (Fig. 2C). There were typical morphological structures of necroptotic cells, such as cytoplasmic hyalinization, loss of cytoplasmic contents, and incomplete cell membranes (indicated by red arrows) in HUVEC after hypoxia (12 h) as observed by TEM (Fig. 2D). Meanwhile, the necroptosis activation markers (p-RIPK3/RIPK3 and p-MLKL/MLKL) gradually increased from hypoxia 6 h (Supplementary S1F, G), and the above results were effectively reversed by Nec-1 pretreatment (Fig. 2E). p-RIPK3 immunofluorescence showed similar results to Western Blot (Fig. 2F). Since necrosomes formed by RIPK1, RIPK3, and MLKL are central to necroptosis [22], we observed an increase in RIPK1-MLKL-RIPK3 interactions after hypoxia (12 h) treatment by co-IP assay (Fig. 2G), suggesting the formation of necrosomes.

In in vivo experiments, immunofluorescence of aortic vessel sections for p-MLKL/CD31 also showed that p-MLKL accumulated more in vascular endothelial cells (Fig. 2H). The expression of p-MLKL/MLKL and inflammatory factors (TNFα, IL-1β) in mouse aortic vessels was significantly increased after 4 weeks of hypoxia (Fig. 2I). These results further confirm that hypoxia promotes the formation of necrosomes and activates the necroptotic pathway, which is responsible for endothelial damage.

### FADD is involved in necroptosis of endothelial cells under hypoxia

**Fig. 3:**
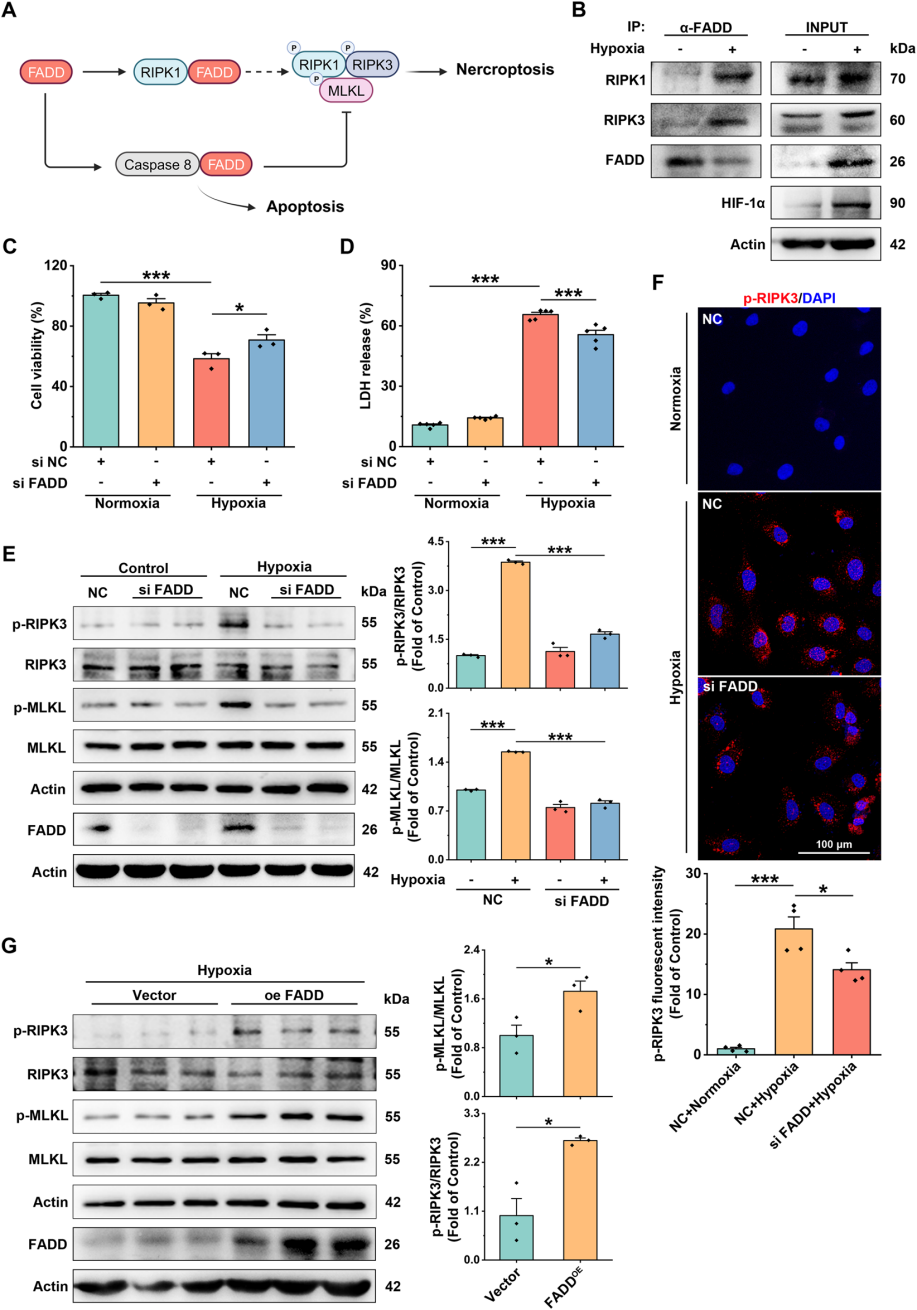
FADD mediates necroptosis in hypoxic endothelial cells. **A** Schematic representation of the mechanism by which FADD regulates apoptosis and necroptosis. **B** HUVEC from normoxic and hypoxic groups were lysed and immunoprecipitated with anti-FADD antibodies, then analyzed by immunoblotting with anti-RIPK3, anti-RIPK1, and anti-FADD antibodies. **C** CCK8 assay to determine changes in cell survival after knockdown of FADD under normoxia and hypoxia, *n* = 3. **D** LDH release assay to determine changes in cytotoxicity after knockdown of FADD under normoxia and hypoxia, *n* = 6. **E** siFADD was used to determine the role of FADD in necroptosis. Representative immunoblot bands and statistical analysis results of HIF-1α, RIPK3, p-RIPK3, MLKL, and p-MLKL in HUVEC of normoxic and hypoxic groups, Actin was used as the reference protein, *n* = 3. **F** Representative confocal images of p-RIPK3 in the corresponding groups and results of statistical analysis, *n* = 4. **G** Representative immunoblot bands and results of statistical analysis of HIF-1α, RIPK3, p-RIPK3, MLKL, p-MLKL in HUVEC of hypoxia group after transfection of FADD overexpression plasmid, Actin was used as the reference protein, *n* = 3. Data are expressed as mean ± SEM. Relevant experiments in this section were performed independently at least three times. *p < 0.05, **p < 0.01, ***p < 0.001.

In previous studies, FADD was reported to regulate necroptosis, with different effects observed in different models (Fig. 3A) [16]. We found that hypoxia (12 h) led to an increase in the interaction of FADD with RIPK1/RIPK3 by co-IP, which suggested that FADD was involved in necroptosis (Fig. 3B). To investigate the specific role of FADD in hypoxia-mediated necroptosis in endothelial cells, we co-incubated siRNA targeting FADD with HUVEC before hypoxia for 48 h. We observed that FADD knockdown significantly restored hypoxia-induced HUVEC cell viability and reduced necrotic cells (Fig. 3C, D). Further, FADD knockdown significantly inhibited the levels of p-RIPK3 and p-MLKL (Fig. 3E, F). On the other hand, we co-incubated the plasmid targeting FADD with HUVEC for 48 h before hypoxia. Western Blot results showed that FADD overexpression significantly increased the levels of p-MLKL and p-RIPK3 (Fig. 3G). These data suggest that FADD plays a vital role in regulating necroptosis in hypoxic ECs.

### Hypoxia promotes FADD SUMOylation, thereby increasing its protein stability

**Fig. 4:**
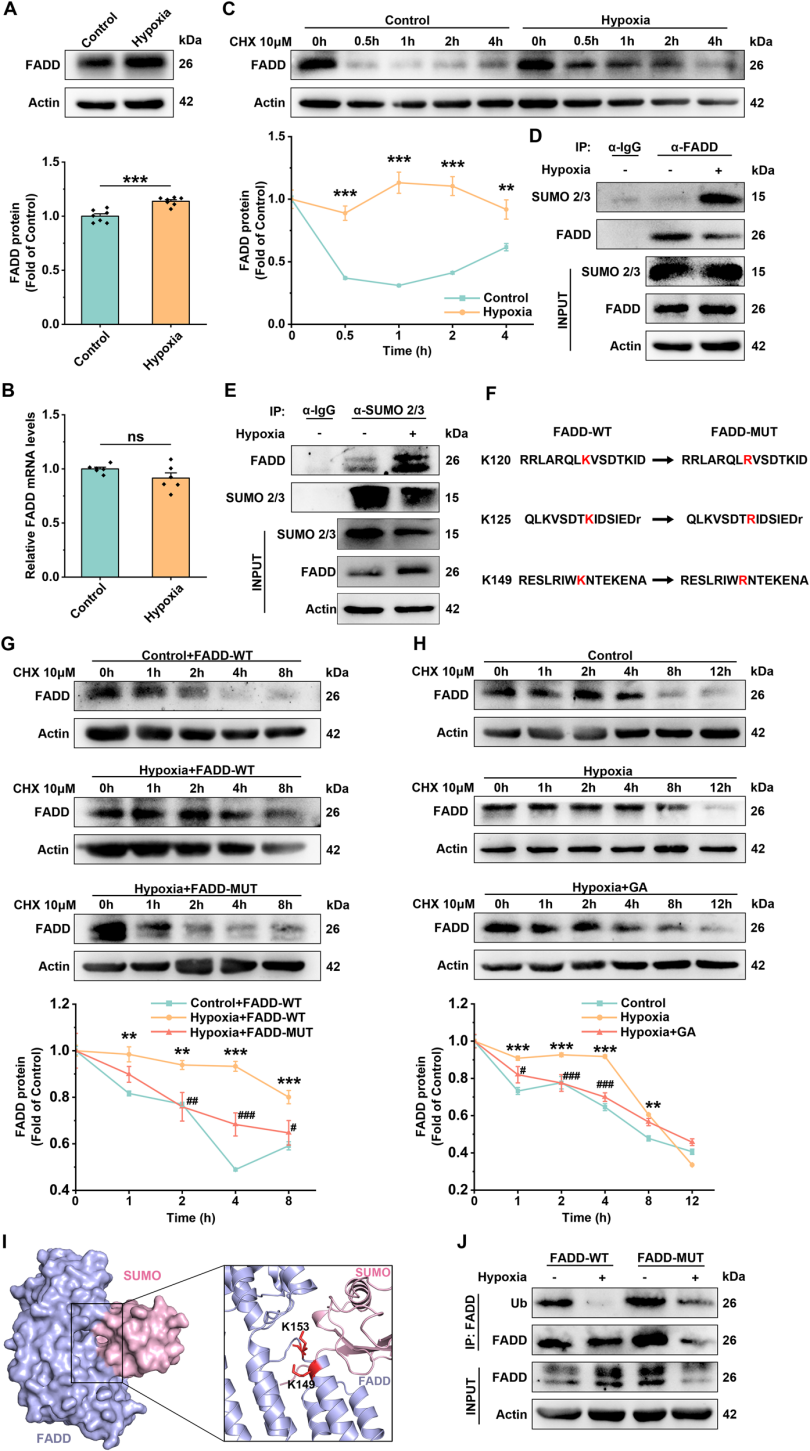
Hypoxia promotes FADD SUMOylation, thereby enhancing its protein stability. **A** Representative immunoblot bands and statistical analyses of FADD in HUVEC after 12 h of hypoxic treatment, Actin was used as the reference protein, *n* = 6. **B** mRNA expression levels of FADD after 12 h of HUVEC hypoxia treatment, with Actin as an internal reference gene, *n* = 7. **C** HUVEC cells were treated with CHX, and cells were collected at different time points after CHX treatment. FADD protein expression levels in HUVEC were analyzed using immunoblotting, and Actin was used as the reference protein. The FADD bands were quantified by ImageJ and the intensity of Actin bands was normalized to calculate the relative. **D** HUVEC from normoxic and hypoxic groups were lysed and immunoprecipitated with anti-FADD antibody, then analyzed by immunoblotting with anti-SUMO2/3, anti-FADD antibody. **E** HUVEC from normoxic and hypoxic groups were lysed and immunoprecipitated with anti-SUMO2/3 antibodies, then analyzed by immunoblotting with anti-SUMO2/3, anti-FADD antibodies. **F** Amino acid sequences of SUMOylation sites K120, K125, K149 and mutated amino acid sequences of FADD. **G** The protein stability of FADD was analyzed in the FADD-OE (WT) and FADD-OE (MUT) groups after hypoxia treatment, and Actin was used as the reference protein. The FADD bands were quantified by ImageJ and normalized to the intensity of Actin bands to calculate the relative values. **H** Representative immunoblot bands of FADD in HUVEC of normoxic and hypoxic groups with and without GA pretreatment after CHX treatment for different time points and Actin was used as the reference protein. The FADD bands of the corresponding groups were quantified by ImageJ and normalized to the intensity of Actin bands to calculate the relative values. **I** Representative structures of FADD and SUMO binary complexes, molecularly docked using pyDockWEB. Where K149 and K153 are ubiquitination sites of FADD. **J** Immunoprecipitation with anti-FADD antibody after lysis of hypoxia-treated FADD-OE (WT) and FADD-OE (MUT) group cells, followed by immunoblotting analysis with antibodies against FADD, anti-Ub. Data are expressed as mean ± SEM. Relevant experiments in this section were performed independently at least three times. *p < 0.05, **p < 0.01, ***p < 0.001.

To elucidate the mechanism underlying FADD-mediated necroptosis, we focused on the changes in the FADD expression. We found that FADD protein levels significantly increased after 12 h of hypoxia in HUVECs (Fig. 4A). Combined with the in vivo experiments, hypoxia for 4 weeks also considerably increased FADD protein levels (Supplementary S2). Interestingly, the mRNA levels of FADD remained unchanged following hypoxia (12 h) (Fig. 4B), suggesting that hypoxia may regulate FADD expression by affecting protein stability rather than transcriptional activity. To explore this hypothesis, we analyzed the rate of protein degradation by using the protein synthesis inhibitor cycloheximide (CHX). As anticipated, hypoxia (12 h) prolonged the half-life of FADD protein (Fig. 4C). Additionally, treatment with the autophagy activator rapamycin did not affect FADD protein levels (Supplementary S3A), further indicating that hypoxia-induced increases in FADD are not mediated by autophagy-related pathways.

Considering the importance of PTMs in regulating protein stability [23], we focused on PTMs of FADD. FADD is known to undergo various PTMs, including ubiquitination, phosphorylation, SUMOylation, etc. Notably, SUMOylation can compete with ubiquitination, potentially influencing protein stability [19, 20]. To this end, we found that hypoxia (12 h) enhanced the interaction between FADD and SUMO2/3 in HUVECs (Fig. 4D, E). To determine whether SUMOylation affects its protein stability, we constructed SUMOylation site mutant plasmids of FADD (three sites mutated to arginine) by targeted mutagenesis (Fig. 4F). We then transfected HUVEC cells with either wild-type FADD (FADD-OE WT) or mutant FADD (FADD-OE MUT) plasmids, confirming successful transfection by co-immunoprecipitation (co-IP) and Western blot analysis (Supplementary S4C-E). The results showed that the FADD-OE (WT) plasmids significantly prolonged the half-life of the FADD protein under hypoxia (12 h). In contrast, the FADD-OE (MUT) plasmids prevented the hypoxia-induced prolongation of the FADD protein half-life (Fig. 4G). Additionally, treatment with the SUMOylation inhibitor ginkgolic acid (GA) reduced FADD protein levels (Supplementary S3B) and shortened the half-life of FADD protein under hypoxia (12 h) (Fig. 4H).

Notably, FADD has multiple ubiquitination binding sites (K149, K153) [18, 20, 24]. We hypothesized that the SUMOylation of FADD might alter its conformation, thereby competitively inhibiting its ubiquitination. As expected, molecular docking results showed that SUMOylated FADD masked its ubiquitination site (K149, K153), effectively inhibiting its ubiquitination (Fig. 4I). Moreover, the ubiquitination levels of FADD were significantly reduced under hypoxia (12 h), whereas the FADD-OE (MUT) plasmids restored the ubiquitination level (Fig. 4J). The above results suggest that hypoxia (12 h) promotes the SUMOylation of FADD in HUVEC, which in turn enhances its protein stability by inhibiting ubiquitination.

### SUMOylated FADD increases its binding to RIPK1 and RIPK3, thereby promoting necroptosis

**Fig. 5:**
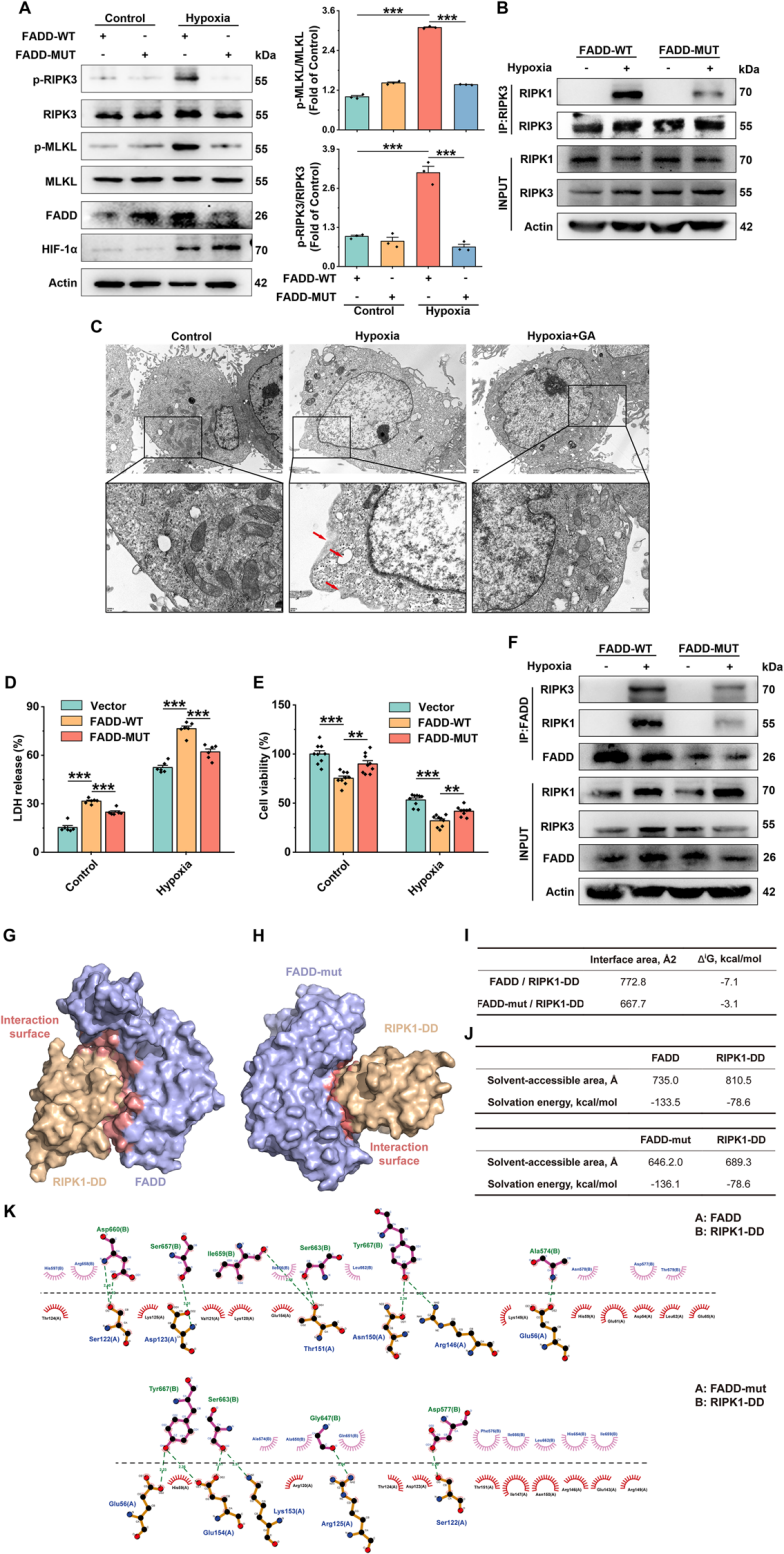
SUMOylated FADD increases its binding to RIPK1 and RIPK3, thereby promoting necroptosis. **A** Representative immunoblot bands of HIF-1α, RIPK3, p-RIPK3, MLKL, p-MLKL and results of statistical analysis in hypoxia-treated cells for 12 h after transfection of HUVEC with the indicated plasmids, Actin was used as the reference protein, *n* = 3. **B** HUVEC were transfected using the indicated plasmids and exposed to normoxic and hypoxic environments, and the cells were lysed and immunoprecipitated with anti-RIPK3 antibody, followed by immunoblotting with anti-RIPK3, anti-RIPK1 antibodies. **C** Representative transmission electron microscopy images of HUVEC exposed to normoxia and hypoxia (with or without GA treatment); Scale bar = 2 μm (merged image) and 500 nm (magnified image), *n* = 3. **D** LDH release assay to determine changes in cytotoxicity after transfection of the indicated plasmids, *n* = 6. **E** CCK8 assay to determine changes in cell survival after transfection with the indicated plasmids, *n* = 9. **F** HUVEC were transfected using the indicated plasmids and exposed to normoxic and hypoxic environments, and the cells were lysed and then immunoprecipitated with anti-FADD antibody and then analyzed by immunoblotting with anti-RIPK1, anti-RIPK3 antibodies. **G**, **H** Representative structures of FADD and RIPK1-DD binary complexes using pyDockWEB for molecular docking. **I**, **J** Results of the interaction surface analysis of FADD and RIPK1-DD complexes. **K** Hydrogen bonds formed during molecular docking simulations of FADD and RIPK1-DD binary complex species. Data are expressed as mean ± SEM. Relevant experiments in this section were performed independently at least three times. *p < 0.05, **p < 0.01, ***p < 0.001.

Previous studies have shown that FADD can participate in necrosome formation to promote necroptosis [25–27]. We speculate that SUMOylation of FADD influences its interaction with RIPK1 and regulates necroptosis. To test this hypothesis, we first elucidate the effect of SUMOylation of FADD on ECs necroptosis. In HUVECs, treatment with FADD-OE (MUT) plasmids significantly suppressed the expression of p-MLKL and p-RIPK3 under hypoxia (12 h) (Fig. 5A, Supplementary S5A). Co-IP results further showed that mutating the SUMOylation site of FADD significantly reduced the formation of the necrosomes (Fig. 5B). Similarly, treatment with the SUMOylation inhibitor GA effectively inhibited hypoxia-induced expression of p-RIPK3/RIPK3 and p-MLKL/MLKL (Supplementary S5B). TEM images showed that GA treatment markedly reverses the number of necroptotic cells (Fig. 5C). FADD-OE(WT) plasmids treatment significantly decreased cell viability and increased cytotoxicity under both hypoxia (48 h) and normoxia. In contrast, FADD-OE (MUT) plasmids did not result in an increase in dead or necrotic cells to the same extent as FADD-WT overexpression (Fig. 5D, E). Meanwhile, GA pretreatment effectively restored cell viability and significantly reduced necrotic cells. (Supplementary S5C, D). These data suggest that inhibiting FADD SUMOylation can suppress necroptosis in ECs.

Next, we explored whether the interaction of FADD with RIPK1/3 depended on its SUMOylation. Co-IP results showed that FADD-OE (WT) plasmids induced the interaction of FADD with RIPK1/3 under hypoxia (12 h), which were markedly reduced by mutating the SUMOylation site of FADD (Fig. 5F). The results suggest that SUMOylation of FADD is essential for the FADD-RIPK1-RIPK3 complex. Molecular docking analyses of the protein crystal structures of FADD and RIPK1 revealed that the complex formed by SUMOylated FADD (FADD-SUMO) and the death domain (DD) of RIPK1 (RIPK1-DD) was more geometrically stable (Fig. 5G, Supplementary S5G). In contrast, the complex formed by docking of FADD mutants and RIPK1 showed more instability (Fig. 5H), with reduced interaction contact area and elevated free energy of binding (Fig. 5I, J), as well as reduction in hydrogen bonds (Fig. 5K). FADD interacted with RIPK1 through the amino acid network of SER122-ASP660, ASP123-SER657, THR151-LLE659, THR151-SER663, ASN150-TYR667, and ARG146-TYR667 (Fig. 5K). Moreover, SUMO facilitated the interaction between FADD and RIPK1 by forming hydrogen bonds with RIPK1 through residues such as GLY64-TYR667, GLN65-SER664, TNR70-ASN593, HIS37-ASP588, etc., which facilitates the interaction between FADD and RIPK1 and stabilizes FADD-SUMO-RIPK1 complexes (Supplementary S5H).

In summary, these results suggest that SUMOylated FADD increases its binding to RIPK1/3, thereby mediating necroptosis.

### SUMOylation inhibitor GA ameliorates hypoxia-induced vascular endothelial injury and inflammatory response

**Fig. 6:**
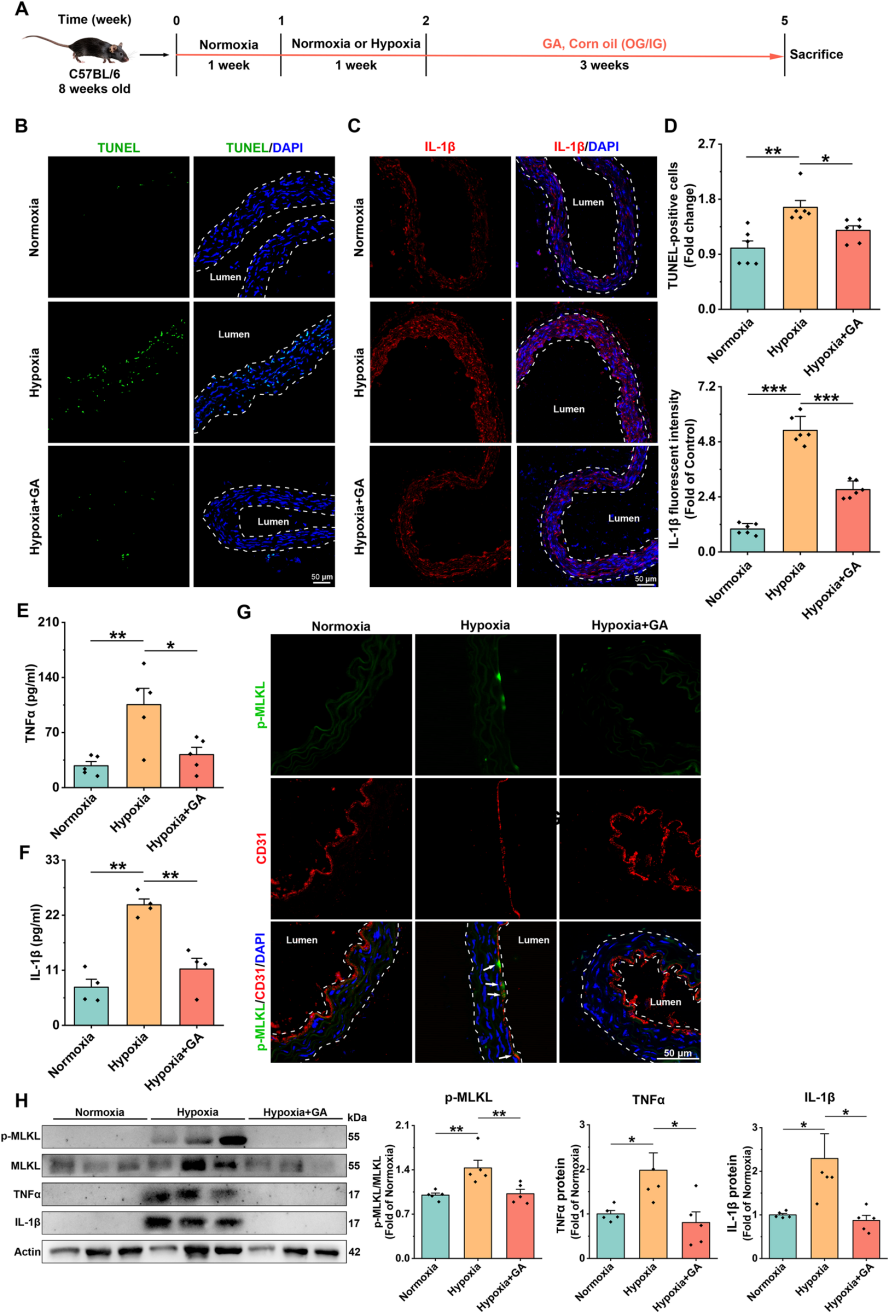
GA inhibits hypoxia-induced vascular endothelial damage and inflammation. **A** Schematic diagram of pharmacological intervention treatment in mice. **B** Representative images of TUNEL staining of mouse aortic vascular sections. Scale bar = 50 μm. **C** Representative images of IL-1β (red) staining of mouse aortic vascular sections. Scale bar = 50 μm. **D** TUNEL staining (*n* = 6) and IL-1β (red) staining (*n* = 6) results of statistical analysis. **E** Serum levels of the inflammatory cytokine TNFα were determined by Elisa assay. *n* = 5. **F** Serum levels of the inflammatory cytokine IL-1β were determined by Elisa assay. *n* = 4. **G** Representative images of CD31 (green) and p-MLKL (red) staining of aortic vascular sections from mice after 4 weeks of hypoxic treatment. Arrows indicate CD31 and p-MLKL co-localized fractions. scale bar = 50 μm. **H** Representative immunoblot bands and statistical analyses of aortic vascular tissues for IL-1β, TNFα, MLKL, and p-MLKL in mice after 4 weeks of hypoxia treatment, Actin was used as the reference protein, *n* = 5. Data are expressed as mean ± SEM. Relevant experiments in this section were performed independently at least three times. *p < 0.05, **p < 0.01, ***p < 0.001.

Having demonstrated that SUMOylation can contribute to hypoxia-induced necroptosis via FADD, we explored whether inhibiting SUMOylation could serve as an effective strategy to mitigate endothelial injury. To assess this, we investigated the protective effects of GA on hypoxia-induced vascular endothelial injury and inflammatory responses in mice. After one week of hypoxia treatment, C57BL/6 mice were administered GA at a concentration of 10 mg/ml by gavage every two days for a duration of three weeks (Fig. 6A) [28]. Consistent with expectations, aortic vascular sections from the GA-treated hypoxic group of mice exhibited less accumulation of TUNEL^+^ cells and IL-1β (Fig. 6B–D). The serum of GA-treated mice showed lower levels of TNFα and IL-1β (Fig. 6E, F). GA significantly reduced hypoxia-induced upregulation of inflammatory factor expression and phosphorylation of MLKL in aortic vascular tissues (Fig. 6G, H, Supplementary S2). Meanwhile, the level of ICAM-1 in the aortic vessels of mice in the GA group was significantly reduced (Supplementary S6). GA reduced organ damage in the liver and spleen (Supplementary S7). These results suggest that GA protects hypoxia-induced vascular endothelial injury by inhibiting necroptosis and inflammatory responses.

## Discussion

**Fig. 7:**
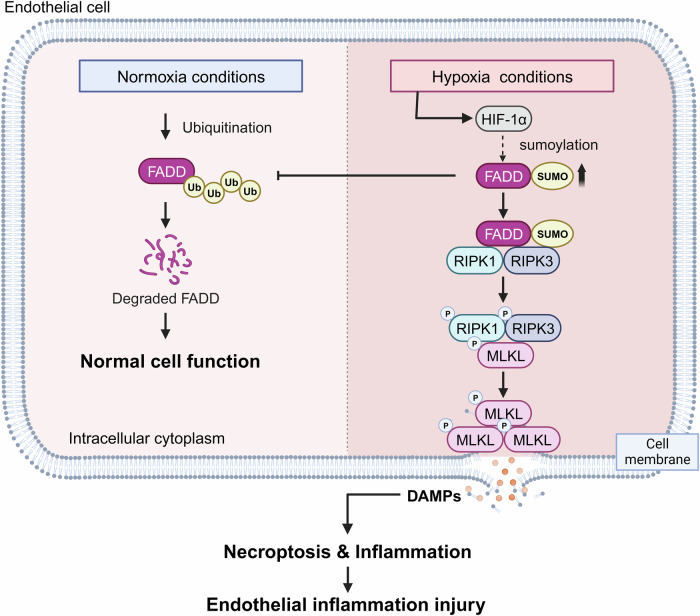
Schematic representation of hypoxia-mediated necroptosis in endothelial cells. Under normoxia, FADD maintains normal cellular physiological functions through ubiquitination-proteasome degradation. Under hypoxic conditions, endothelial cells increase SUMOylation of FADD to inhibit its ubiquitination level, which increases the protein stability of FADD as well as promotes the binding of FADD to RIPK1/3, inducing the formation of necrosomes, and promotes necroptosis and inflammatory injury of endothelial cells.

Hypoxia can be defined as a physiological or pathological state in which there is a lack of sufficient oxygen supply at the tissue level to meet the needs of the cells or tissues [1, 29]. It is present in numerous diseases, such as atherosclerosis, where an imbalance between oxygen supply and demand results in high-fat arterial wall lesions and plaque formation, increased lipid accumulation, and inflammatory responses [6–8]. In contrast, the specific mechanisms by which hypoxia induces vascular lesions and necroptosis activation are not known. Here, we found that necroptosis may be an essential cause of hypoxic endothelial injury, while SUMOylated FADD plays a vital role (Fig. 7), which contributes to provide new and promising targets of hypoxic diseases treatment.

Necroptosis, an emerging programmed cell death pathway, is involved in and regulates multiple diseases, such as cancer, neurological diseases, and retinal diseases [30–32]. Nevertheless, there is a paucity of studies that have investigated the role of necroptosis in hypoxia-mediated vascular endothelial injury. Our in vivo and in vitro experiments demonstrated that hypoxia induces necroptosis in vascular endothelial cells (ECs) by promoting the formation of the necrosome, which ultimately leads to endothelial dysfunction. Studies have shown that necroptosis is an inflammatory cell death, leading to the production of inflammatory cytokines [9, 10]. Here, we demonstrated that inflammatory factors were produced in vascular tissues as well as the serum of mice after hypoxia. Meanwhile, inflammatory factors were highly expressed in the ECs and smooth muscle cells, but p-MLKL was only expressed in vascular ECs (Figs. 1B, 2H, I). This may be due to the release of inflammatory cytokines after necroptosis of ECs, which induces the activation of inflammatory signals in smooth muscle cells. Previous studies have demonstrated that pro-inflammatory factors in the inflammatory microenvironment, such as TNFα, are the classical promoters of necroptosis [9, 10]. However, the present study did not delve deeper into the predominant factors of hypoxia-induced necroptosis in the endothelium, which requires further exploration.

As a crucial bridging protein, FADD has been identified as a master regulator of apoptosis, necroptosis, and inflammation, depending on cell type and context [16]. Most studies have reported the inhibitory effect of FADD on necroptosis [16]. Interestingly, the present study demonstrates that FADD plays a pro-necroptotic role in hypoxia-mediated necroptosis of endothelial cells [14–16]. The conflicting results observed in these studies may be attributed to the activity of caspase 8 within the cells. We observed a decrease in caspase 8 activity under hypoxic conditions (Supplementary S1B), which resulted in a shift in FADD to form a complex with RIPK1/RIPK3, thereby promoting necroptosis.

SUMOylation plays an important role in hypoxic diseases, being an essential PTM of proteins that can regulate protein-protein interactions, protein stability, and cellular localization [33]. In a model of pulmonary arterial hypertension constructed in mice with chronic hypoxia, the FIS1 deSUMOylation-SUMOylation transition in pulmonary endothelium is an intrinsic pathogenesis of hypoxic PH [33]. Similarly, FADD can undergo SUMOylation after hypoxia treatment, but the effect of SUMOylation of FADD on its protein function and its effect on hypoxia-induced endothelial damage was unknown. In the present study, we demonstrate that SUMOylation of FADD increases protein stability while recruiting RIPK1/RIPK3 to promote necrotic apoptosis under hypoxia.

The effects of ubiquitination and SUMOylation on protein function are controversial due to the shared nature of lysine protein residues between ubiquitinated and SUMOylated proteins [34]. A recent study has indicated that the SUMOylation of HIF-1α induced by hypoxia targets HIF-1α for degradation through the von Hippel-Lindau protein-mediated UPS [35]. In this case, there is a competition between SUMOylation and ubiquitination in FADD, rather than a collaborative process, resulting in a notable increase in its protein stability. Our molecular docking results provide an explanation for this observation, as the SUMOylated FADD has masked the two ubiquitination sites. Both SUMOylation and ubiquitination require E3 ligases to function [18, 19], and a shortcoming of this study is that it did not investigate which specific E3 ligase functions in SUMOylation processes.

We herein identified a crucial role for SUMOylated FADD in hypoxia-induced necroptosis. So far, the importance of SUMOylation has been established in multiple cellular death patterns, including apoptosis, autophagy, senescence, and pyroptosis [36–40]. In pyroptosis, SUMOylation of NLRP3 restrains inflammasome activation [37]. We now report an additional regulation of necroptosis by SUMOylation, and SUMOylation of FADD promotes its binding to RIPK1/RIPK3, thereby facilitating necroptosis. We further show that inflammatory injury as well as necroptosis of aortic vascular endothelium in hypoxic mice can be alleviated by inhibiting SUMOylation. Therefore, we suggest the possibility of inhibiting FADD SUMOylation as a therapeutic strategy for reducing hypoxic disease pathogenesis by inhibiting vascular inflammation and ECs necroptosis. To our knowledge, our finding is the first demonstration of the critical role of SUMOylation in necroptosis in the vascular system. One major limitation of the present study is that we omitted investigations into the relationship between SUMOylation and hypoxia, which will be focused on in our future studies.

Nec-1 is a well-known small-molecule inhibitor of necroptosis and can attenuate a wide range of injuries caused by necroptosis in animal experiments [41, 42]. However, a safe and effective drug to block necroptosis is still lacking in the clinic. Ginkgolic acid is an alkyl phenolic constituent extracted from the Ginkgo biloba [28]. Previous studies have shown that GA possesses numerous biological activities, including anti-inflammatory, anti-tumor, and anti-bacterial [43, 44]. Here, we demonstrated that GA inhibited necroptosis, inflammatory responses, and endothelial damage in vascular tissues. Since GA can also inhibit SUMOylation of proteins, we did not investigate in depth whether the effect of GA in inhibiting necroptosis originated from its anti-inflammatory or SUMOylation inhibition effect.

In conclusion, our study reveals the role of SUMOylation of FADD in hypoxia-mediated necroptosis in ECs. This study on necroptosis also provides new insights into therapeutic targets for endothelial injury. GA could play an essential role as a necroptosis inhibitor in the clinical treatment of hypoxic diseases.

## Materials and methods

### Animal studies

Healthy male C57BL/6 mice (8–10 weeks old, weighing 15–20 g) were provided by the Animal Centre of Chongqing Medical University. All mice were housed in regulated experimental animal facilities with no more than 5 mice per cage, equipped with light (12 h light and 12 h dark environmental cycle), suitable temperature (24 ± 2 °C), humidity (50 ± 10%), and adequate food and water.

Block pseudo-randomization was used for experimental group allocation. The investigators were blinded to grouping assignments. For the hypoxia intervention, mouse cages were placed in a hypoxic laboratory mouse incubator (PH-AM Wuxi Bodho). The hypoxic chamber was filled with nitrogen to replace oxygen to reduce the oxygen concentration, and the oxygen concentration of the hypoxic chamber was set at 10%. The mice were rapidly euthanized by intraperitoneal injection of 150 mg/kg sodium pentobarbital at the indicated time points, and tissues were quickly collected for histological studies.

### Cell culture and hypoxia intervention

Human Umbilical Vein Endothelial Cells (HUVEC) were acquired from ScienCell Research Laboratories, Inc. (USA) and cultured in DMEM medium with 10% fetal bovine serum (FBS) (Adamas Life, China). To establish hypoxic conditions for cell culture, the Whitely H35 HEPA Hypoxia Workstation (DonWhitley Scientific Limited, UK) was used, with gas levels set to 1% O_2_, 5% CO_2_, and 94% N_2_. HUVECs were incubated under either normoxic or hypoxic conditions, following standard culture protocols, and experimental reagents were added at designated time points for each experimental setup.

### Western blot

Protein samples were collected by lysing cells or vascular tissues with RIPA lysis solution (Beyotime, China). Aliquots of denatured protein samples were separated by SDS-PAGE gel and transferred to the PVDF membrane (Millipore, United States). After being blocked with TBST solution containing 5% BSA, the membranes were incubated with primary antibodies at 4 °C overnight. Subsequently, the membranes were incubated with secondary antibodies and exposed to chemiluminescence imaging system (UVP) development using an ECL kit (Biosharp, China). Protein levels were analyzed by ImageJ software.

The following primary antibodies were used:

Anti-β-Actin (HRP-66009, Proteintech Group, United States), anti-RIPK3 (17563-1-AP, Proteintech Group), anti-RIPK1 (17519-1-AP, Proteintech Group), anti-MLKL (66675-1-Ig, Proteintech Group), anti-FADD (14906-1-AP, Proteintech Group), anti-SUMO2/3 (11251-1-AP, Proteintech Group), anti-p-RIPK3 (ab209384, Abcam, United Kingdom), anti-TNF-α (SC-1351, Santa Cruz Biotech, United States), anti-IL-1β (A17361, Santa Cruz Biotech), anti-HIF-1α (AF1009, Affinity Biosciences), anti-p-MLKL (AF7420, Affinity Biosciences, United States), VCAM (A0279, ABclonal Technology), ICAM (A5597, ABclonal Technology).

### Immunofluorescence assay

For immunofluorescence of cells, HUVEC cells were seeded into 24-well plates containing 10 mm × 10 mm slides. The cells were fixed with 4% PFA and blocked with 5% BSA. Subsequently, the cells were incubated with the primary antibody at 4 °C overnight, the secondary antibody at room temperature for 1 h, and DAPI staining for 10 min.

For immunofluorescence of tissue sections, tissues collected from mice were fixed with 4% PFA, and paraffin-embedded sections were performed. After dewaxing and hydration, the paraffin sections were antigenically repaired with 10 mM citrate buffer, blocked with 5% BSA, and incubated with primary antibody overnight at 4 °C. After being washed by PBST, it was incubated with a secondary antibody at room temperature and stained with DAPI for 5 min.

Fluorescence images were collected using a confocal microscope system (Leica, Japan) using 20× or 40× objective lenses.

The following primary antibodies were used:

Anti-p-RIPK3 (ab209384, Abcam), anti-HIF-1α (A22041, ABclonal Technology), anti-p-MLKL (AF7420, Affinity Bioscience), anti-HIF-1α (AF1009, Affinity Biosciences), anti-IL-1β (A17361, Santa Cruz Biotech), anti-CD31 (ab182981, Abcam).

### RT-qPCR

Total RNA was extracted using the Trizol method, and RNA concentration was measured using an Implen Ultra-Micro Spectrophotometer. cDNA was prepared using Evo M-MLV Mix Kit with gDNA Clean for qPCR (Accurate Biology, China). cDNA quantification for each sample was performed using SYBR® Green Realtime Master qPCR Mix (Accurate Biology, China) for Real-time PCR. Quantitative cDNA was subjected to Real-time PCR using SYBR® Green Realtime Master qPCR Mix (Accurate Biology, China). Data were collected using Bio-Rad CFX Maestro (Bio-Rad, United States) software and analyzed using the 2^-ΔΔCt method.

The gene-specific primer sequences used for PCR are shown in Table 1.

**Table 1:** Oligonucleotide primer sequences used for quantitative real-time PCR.

Gene	Primer sequence (5′–3′)
*HIF-1α*	F: GAACGTCGAAAAGAAAAGTCTCG
	R: CCTTATCAAGATGCGAACTCACA
*IL-1β*	F: ATGATGGCTTATTACAGTGGCAA
	R: GTCGGAGATTCGTAGCTGGA
*TNFα*	F: CCATGTTGTAGCAAACCCTCAAG
	R: AAGAGGACCTGGGAGTAGATGAG
*FADD*	F: GTGGCTGACCTGGTACAAGAG
	R: GGTAGATGCGTCTGAGTTOOAT
*Caspase 3*	F: TGAGCCATGGTGAAGAAGGAATAA
	R: CCCGGGTAAGAATGTGCATAAAT
*Caspase 8*	F: TCAACAAGAGCCTGCTGAAGATA
	R: GGAGAGTCCGAGATTGTCATTAC
*c-Flip*	F: ATGGCAGAGATTGGTGAGGATTT
	R: GCTCCTTGAACAGACTGCTTGTA
*β-Actin*	F: CATGTACGTTGCTATCCAGGC
	R: CTCCTTAATGTCACGCACGAT

### siRNA interference

FADD siRNA was purchased from Tsingke Biotech (China) and Lipofectamine™ 2000 from Invitrogen (United States). According to the instruction manual, siRNA was transfected into HUVEC cells using Lipofectamine™ 2000.

The siRNA sequences used for interference are shown in Table 2.

**Table 2:** siRNA primer sequences used for siRNA interference.

Gene	Primer sequence (5′-3′)
*FADD*	F: GCGAGCUGACCGAGCUCAA
	R: UUGAGCUCGGUCAGCUCGC

### Plasmid transfection

The K120, K125, and K149 mutant plasmids of FADD (lysine mutated to arginine) were purchased from Shanghai Biotechnology, and the Neofect® DNA transfection reagent was purchased from Genomtech (China).

According to the instruction manual, HUVEC cells were transfected with Neofect® DNA transfection reagent for the plasmid transfection.

### Immunoprecipitation

Protein samples were collected by lysing cells with RIPA lysis solution. Protein A+G magnetic beads (MCE) were incubated with the indicated antibodies for 4 h at 4 °C, and then cell lysates were incubated with magnetic beads overnight at 4 °C to capture the immune complexes. After three washes with PBST, the magnetic beads were boiled in SDS-PAGE uploading buffer at 99 °C for 10 min, and the samples were separated by SDS-PAGE and transferred to PVDF membranes for immunoblotting analysis with the indicated antibodies.

### Analysis of cytotoxicity and viability

HUVEC cells were inoculated in 96-well plates at 2000 cells per well. Necrotic cells were determined by detecting cellular LDH release using the LDH Cytotoxicity Assay Kit (Beyotime, China) according to the instruction manual. Cell viability was detected using Enhanced Cell Counting Kit 8 (WST-8/CCK8) (Elabscience, China) according to the instruction manual.

### TUNEL tissue staining

TUNEL staining was performed using the One Step TUNEL Apoptosis Assay Kit (Beyotime, China) according to the instruction manual.

### Transmission electron microscopy (TEM)

HUVEC cells were fixed with 3% glutaraldehyde, dehydrated, and embedded in Epon 812 to make ultrathin sections (60–90 nm). After staining using uranyl acetate and lead citrate, they were observed by transmission electron microscopy (JEM-1400FLASH, Japan).

### Enzyme-linked immunosorbent assay (ELISA)

Serum levels of cytokines IL-1β and TNFα were measured using Mouse IL-1 beta ELISA Kit (KE10003, Proteintech Group) and Mouse TNF-alpha ELISA Kit (KE10002, Proteintech Group) according to the instruction manual.

### Data and statistical analysis

Sample size calculation was not conducted, while sample sizes were based on previous studies using similar analysis [45]. Data in this study are expressed as mean ± SEM, and all experiments were repeated at least three times to ensure experimental reproducibility. Data were statistically analyzed using Prism 8.0 (GraphPad Software, USA) using the Two-tailed unpaired t-test or one-way ANOVA. Differences were considered statistically significant if *p* < 0.05.

## Supplementary information

Supplementary Materials

Supplemental Materials

## Data Availability

Data will be made available on request.
