# Supplementary material for: Hypoxia-mediated SUMOylation of FADD exacerbates endothelial cell injury via the RIPK1-RIPK3-MLKL signaling axis
Source: Cell Death Dis. 2025 Feb 21;16(1):121. doi: 10.1038/s41419-025-07441-2 (PMC11845712; doi:10.1038/s41419-025-07441-2)
Supplement: Supplementary file 1 — Supplementary Materials [file 41419_2025_7441_MOESM1_ESM.pdf]

Supplementary Materials

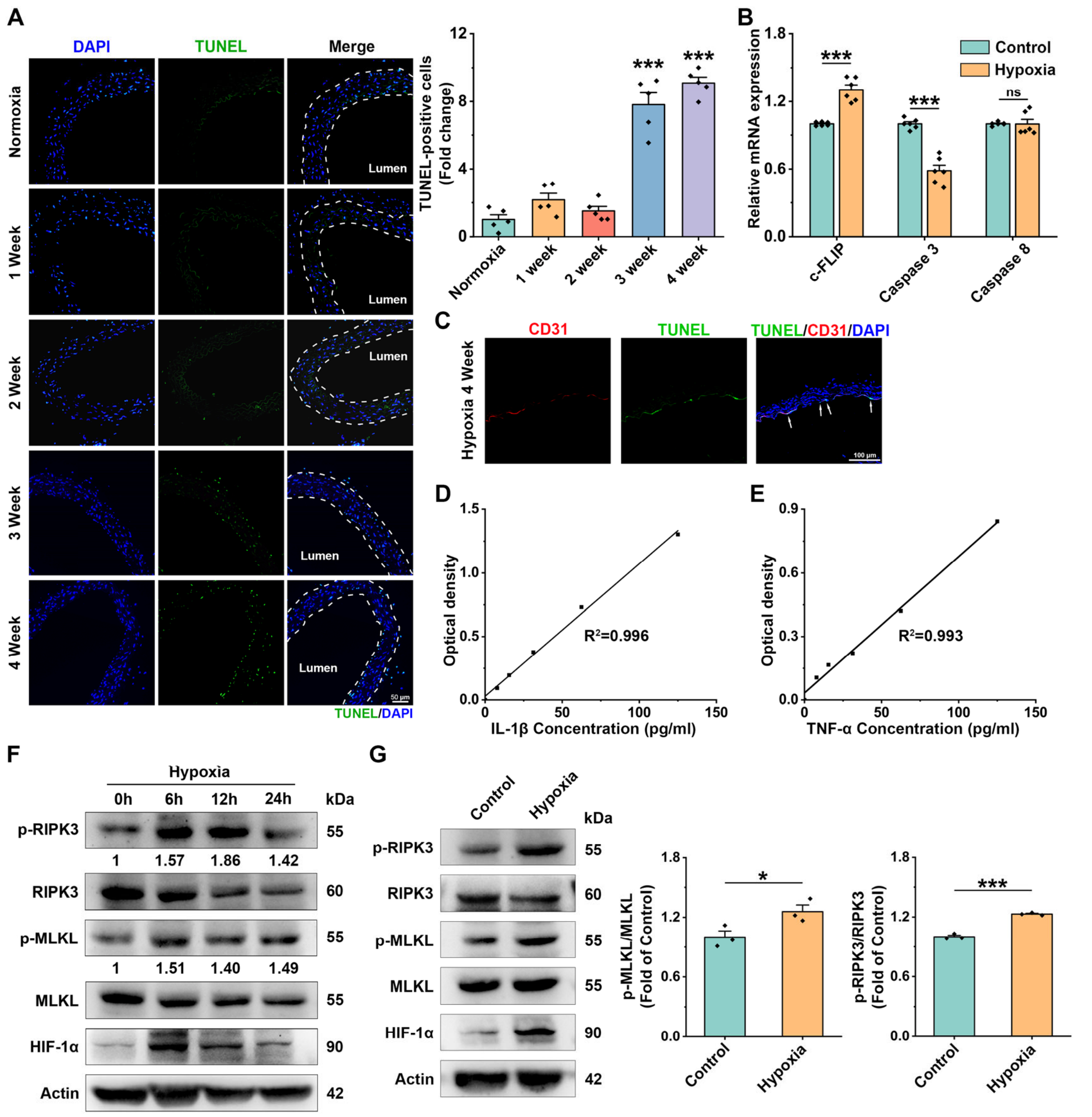

Supplementary S1.

(A) Representative images of TUNEL staining of aortic vessel sections from mice with different times of hypoxia and results of statistical analysis. Scale bar = 50 μm, n=5.

(B) Changes in mRNA expression levels of c-FLIP, Caspase 3, and Caspase 8 after 12h of hypoxia treatment in HUVEC cells, with Actin as an internal

reference gene, n=6.

(C) Representative images of TUNEL (green) and CD31 (red) staining of mouse aortic vascular sections of hypoxic treatment. Arrows indicate CD31 and TUNEL co-localized fractions. Scale bar = 100 μm.

(D) Standard curve for the determination of serum inflammatory factor IL-1β levels by ELISA.

(E) Standard curve for the determination of serum inflammatory factor IL-1β levels by ELISA.

(F) Immunoblot bands of HIF-1α, RIPK 3, p-RIPK3, MLKL, and p-MLKL from hypoxia-treated HUVEC cells at different time intervals, Actin was used as the reference protein.

(G) Representative immunoblot bands and statistical analyses of HIF-1α, RIPK3, p-RIPK3, MLKL, and p-MLKL in hypoxia-treated HUVEC cells for 12 h. Actin was used as the reference protein, n=3.

Data are expressed as mean ± SEM. Relevant experiments in this section were performed independently at least three times. *$p<0.05$, ***$p<0.001$.

A

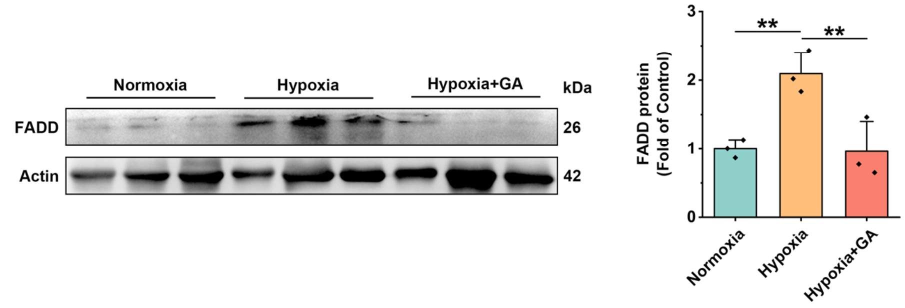

Supplementary S2.

(A) Representative immunoblot bands and statistical analysis of FADD in aortic vascular tissues of mice 4 weeks after hypoxia and GA treatment, Actin was used as the reference protein, n=3.

Data are expressed as mean ± SEM. Relevant experiments in this section were performed independently at least three times. ***$p<0.01$.*

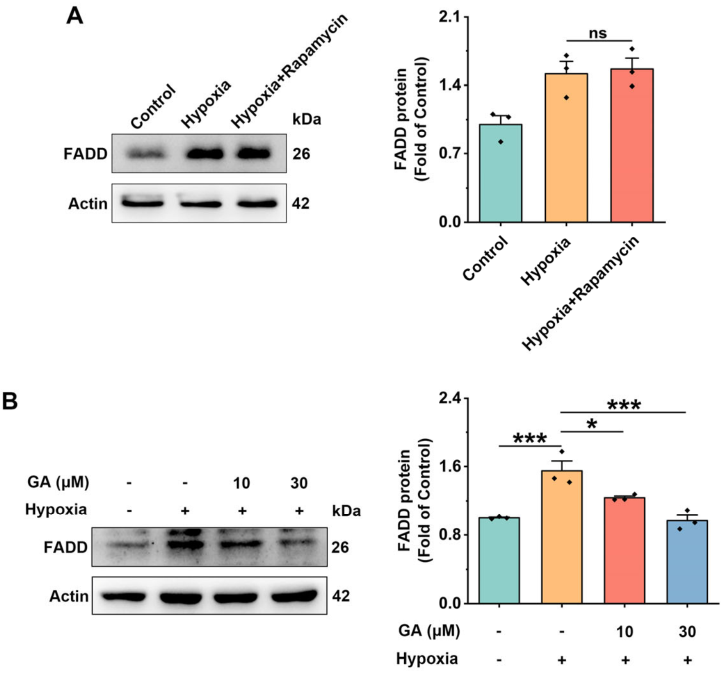

Supplementary S3.

(A) Representative immunoblot bands of FADD in HUVEC cells pretreated with 1 μM Rapamycin for 1 h after hypoxia for 12 h. Actin was used as the reference protein. n=3.

(B) Representative immunoblot bands of FADD in HUVEC cells pretreated with 10 μM and 30 μM GA for 1 h after hypoxia for 12 h. Actin was used as the reference protein. n=3.

Data are expressed as mean ± SEM. Relevant experiments in this section were performed independently at least three times. **p<0.05, ***p<0.001.*

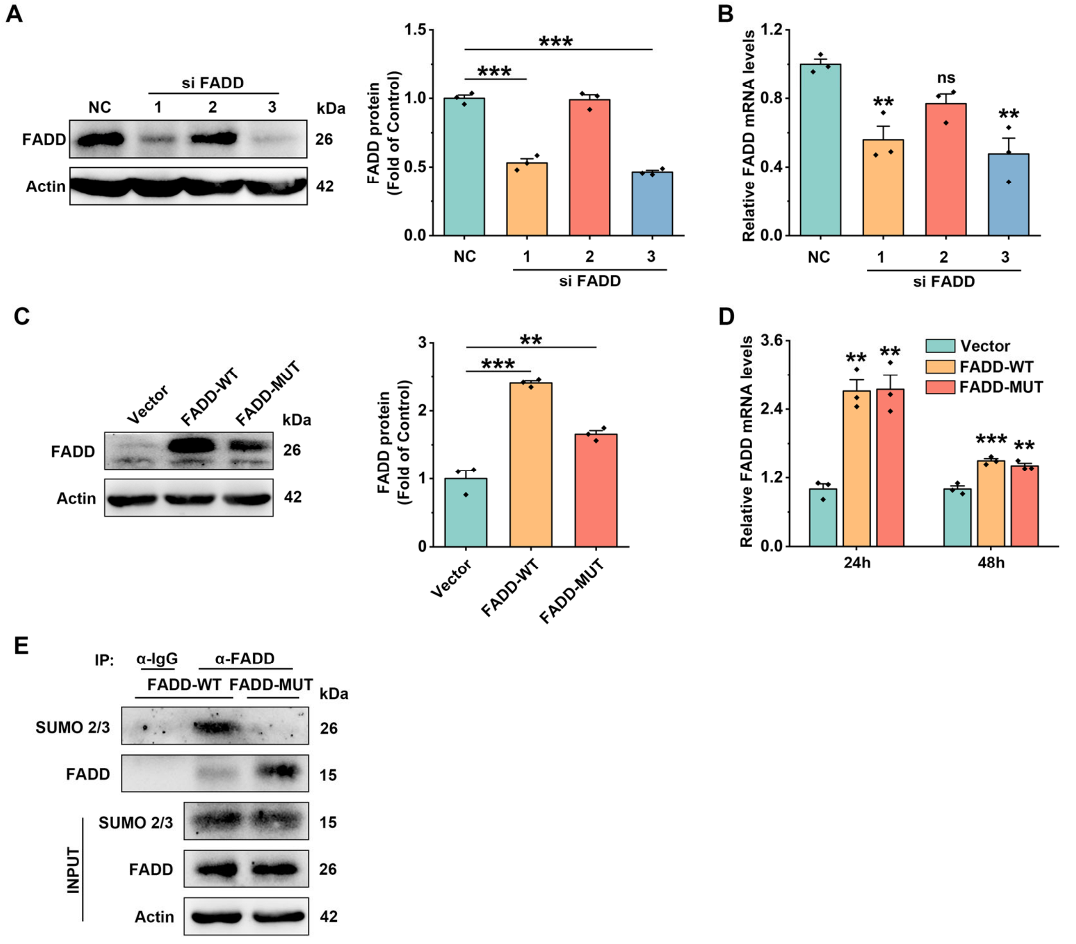

Supplementary S4.

(A) Representative immunoblot bands of FADD after HUVEC transfection of siRNA for 48h, Actin was used as the reference protein, n=3.

(B) mRNA levels of FADD 24h after HUVEC transfection of siRNA, with Actin as an internal reference gene, n=3.

(C) Representative immunoblot bands of FADD after HUVEC transfection of FADD plasmid for 48h, Actin was used as the reference protein, n=3.

(D) mRNA levels of FADD after HUVEC transfection of FADD plasmid 24h, 48h, with Actin as an internal reference gene, n=3.

(E) HUVEC transfected with FADD plasmid were lysed and immunoprecipitated

with anti-FADD antibody, then analyzed by immunoblotting with anti SUMO2/3, anti-FADD antibody.

Data are expressed as mean ± SEM. Relevant experiments in this section were performed independently at least three times. *$**p<0.01$, $***p<0.001$.*

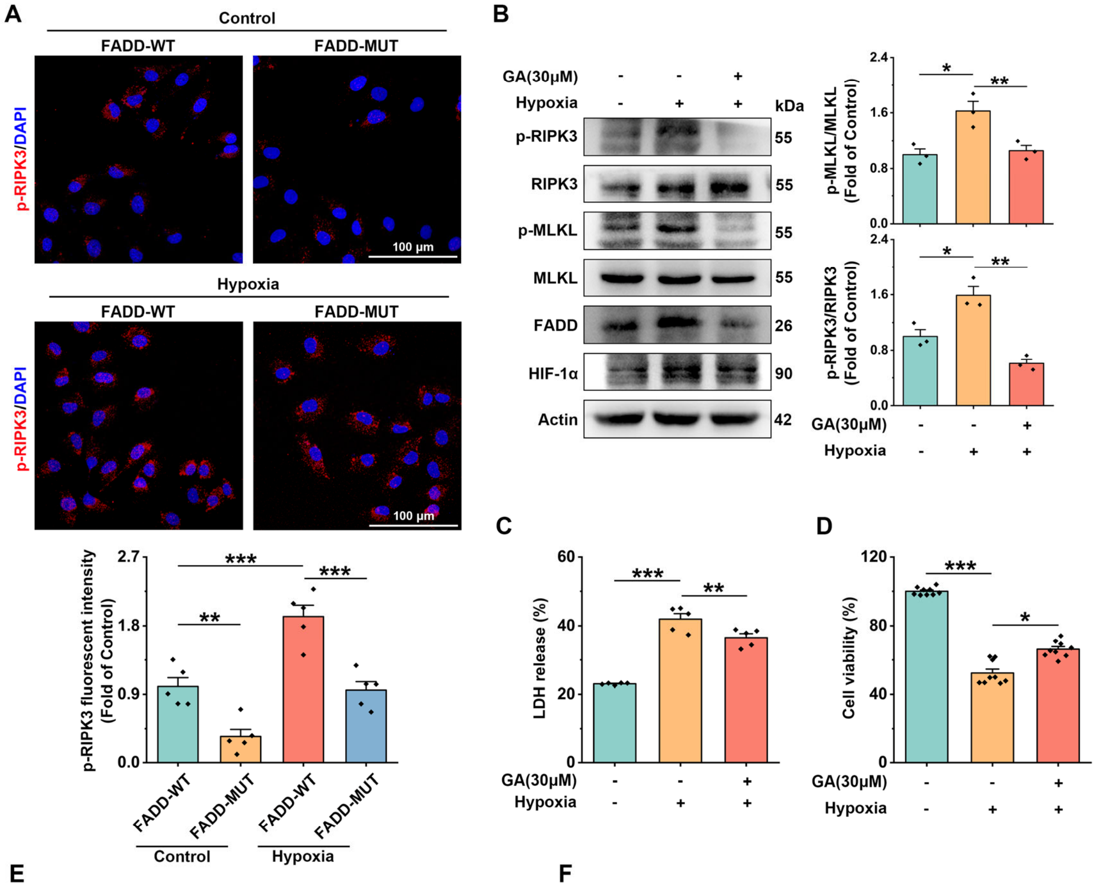

E

Electrostatics	Desolvation	VdW	Total	Rank
-13.338	-15.123	41.821	-24.279	1
-19.072	-9.122	36.476	-24.546	2
-14.070	-13.472	39.958	-23.546	3
-23.036	-0.617	3.962	-23.257	4
-32.072	11.605	-16.548	-22.123	5
-25.763	-0.005	34.660	-22.351	6
-36.492	12.706	22.739	-21.512	7
-20.737	-5.138	40,715	-21.803	8
-20.804	3.091	16.425	-21.071	9
-11.243	-16.659	59.907	-21.912	10

F

Electrostatics	Desolvation	VdW	Total	Rank
-25.211	-1.568	-3.835	-27.163	1
-30.659	-1.661	72.525	-25.068	2
-23.534	0.067	-7.371	-24.203	3
-13.944	-18.328	83.226	-23.950	4
-25.812	0.904	13.262	-23.582	5
-41.827	17.231	8.531	-23.743	6
-12.415	-18.066	67.188	-23.762	7
-31.945	8.388	-0.421	-23.599	8
-22.332	-7.551	69.685	-22.914	9
-31.329	6.804	24.732	-22.051	10

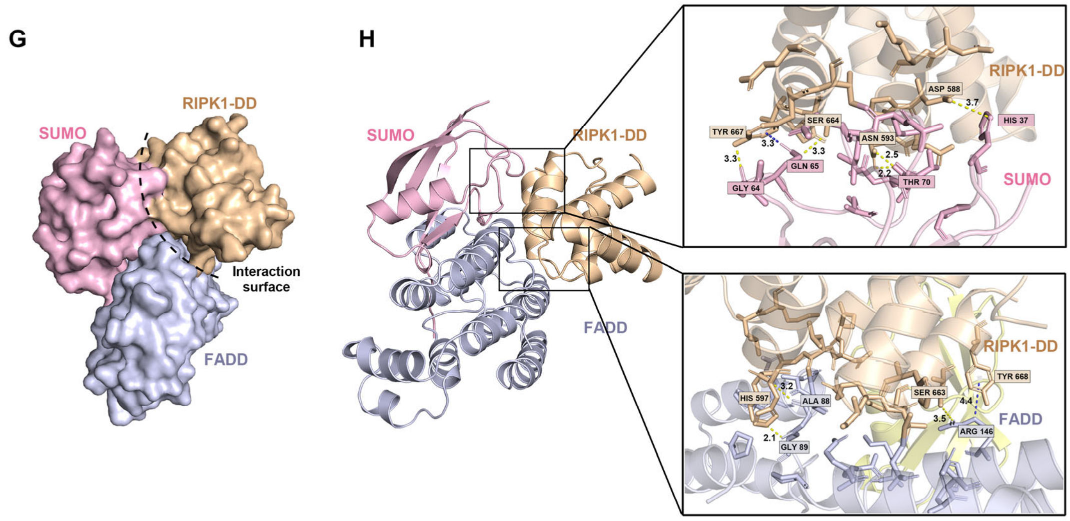

Supplementary S5.

(A) Representative confocal images of p-RIPK3 in the corresponding groups and results of statistical analysis, n=5.

(B) Representatives immunoblot bands of HIF-1α, RIPK3, p-RIPK3, MLKL, p-MLKL in hypoxia-treated cells for 12 h after 1 h of pre-treatment of HUVEC with GA and statistical analyses, Actin was used as the reference protein, n=3.

(C) LDH release assay to determine changes in cytotoxicity following GA addition under hypoxia, n=5.

(D) CCK8 experiments to determine changes in cell survival following the addition of GA under hypoxia, n=9.

(E) Plot of top ten structural affinity values for molecular docking scores of FADD and RIPK1-DD binary complexes.

(F) Plot of top ten structural affinity values for molecular docking scores of FADD-MUT and RIPK1-DD binary complexes.

(G) Representative structure of the ternary complex of FADD-SUMO with RIPK1-DD using pyDockWEB for molecular docking

(H) Hydrogen bonds formed during molecular docking simulations of FADD-SUMO and RIPK1-DD ternary complexes.

Data are expressed as mean ± SEM. Relevant experiments in this section were performed independently at least three times. *$p<0.05$, **$p<0.01$, ***$p<0.001$.

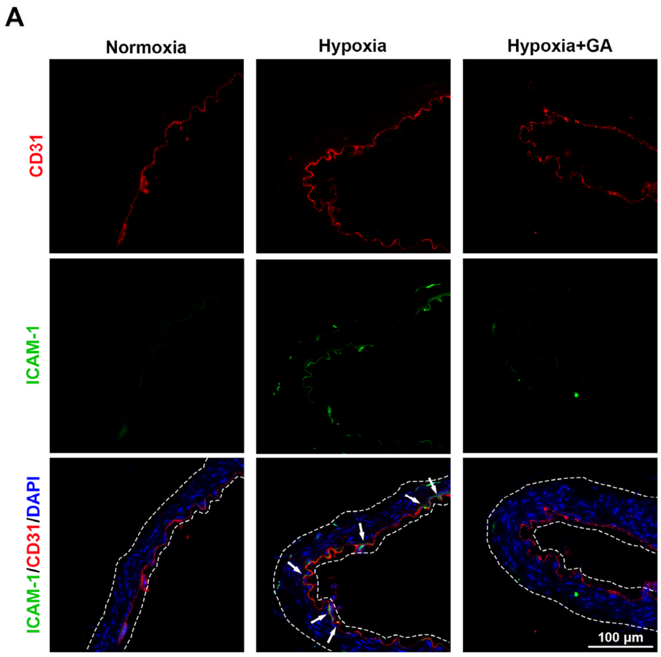

Supplementary S6.

(A) Representative images of CD31 (red) and ICAM-1 (green) staining of aortic vascular sections from mice after 4 weeks of hypoxic treatment. Arrows indicate CD31 and ICAM-1 co-localized fractions. Scale bar = 100 μm.

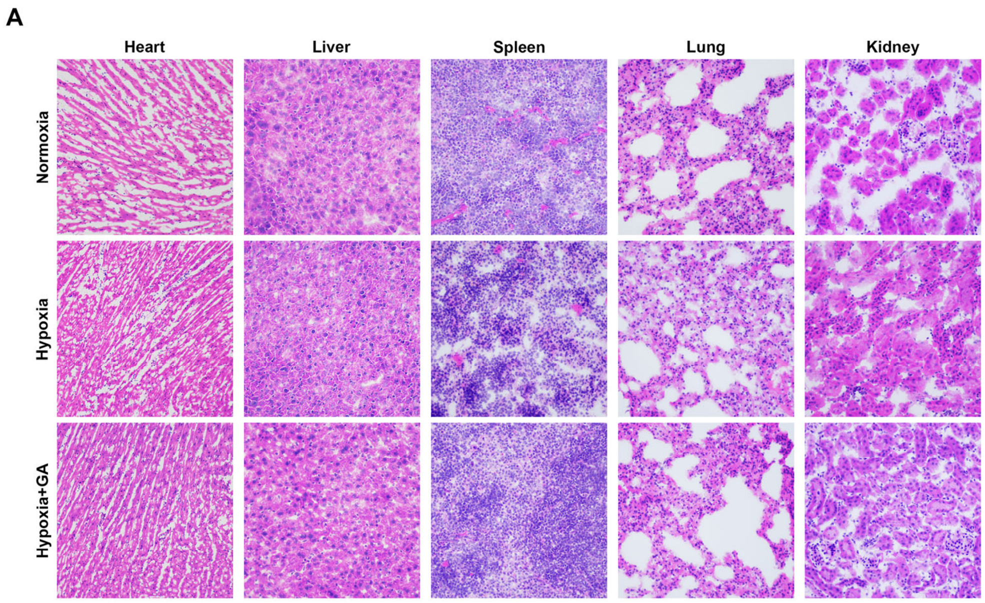

Supplementary S7.

HE stains of mouse heart, liver, spleen, lung, and kidney sections.
