# Supplementary material for: Hypoxia-mediated SUMOylation of FADD exacerbates endothelial cell injury via the RIPK1-RIPK3-MLKL signaling axis
Source: Cell Death Dis. 2025 Feb 21;16(1):121. doi: 10.1038/s41419-025-07441-2 (PMC11845712; doi:10.1038/s41419-025-07441-2)

Supplementary S5 Original full length western blots for Fig 1

Fig 1F

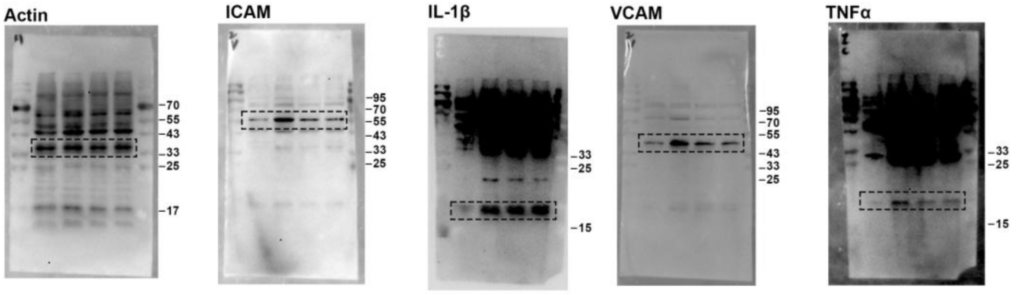

Supplementary S6 Original full length western blots for Fig 2

Fig 2D

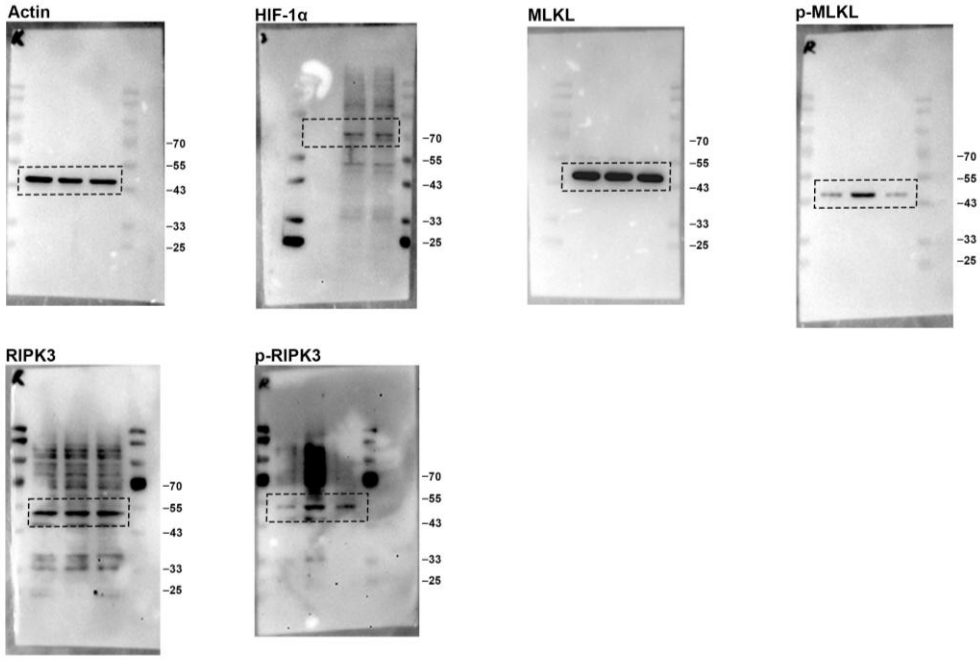

Fig 2G

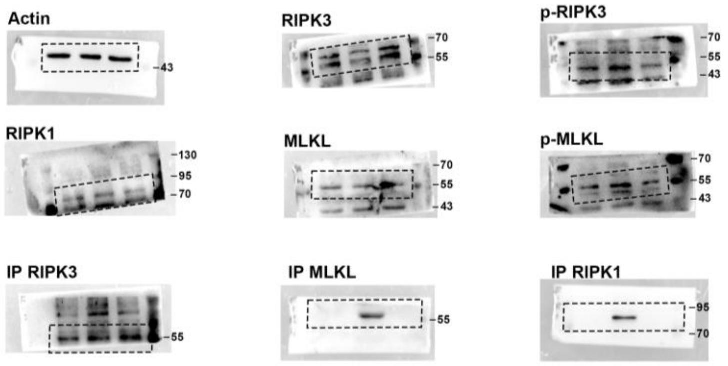

Fig 2I

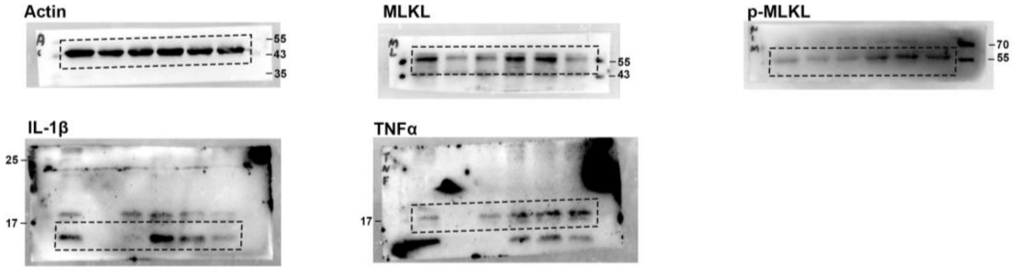

Supplementary S7 Original full length western blots for Fig 3

Fig 3E

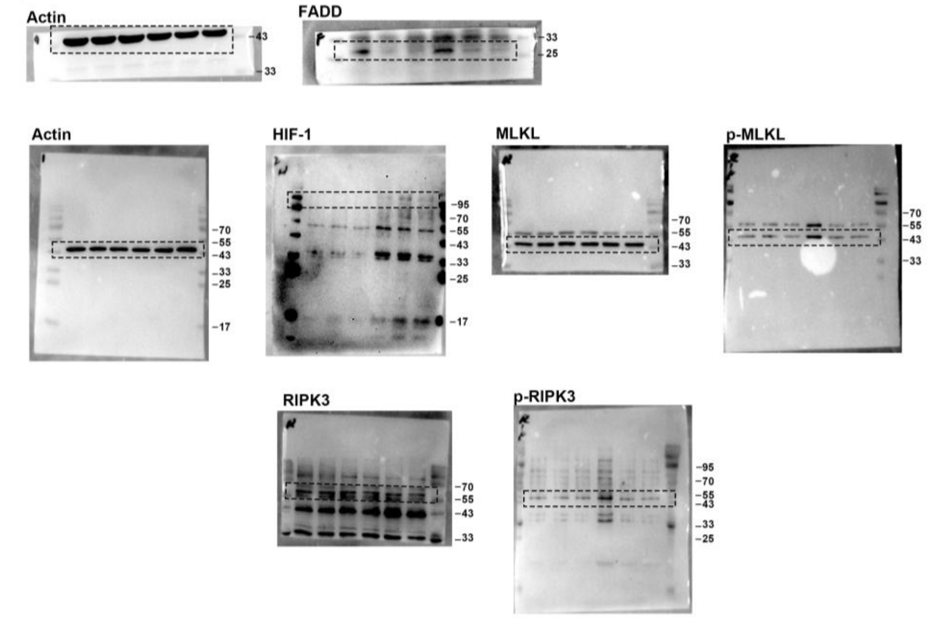

Fig 3G

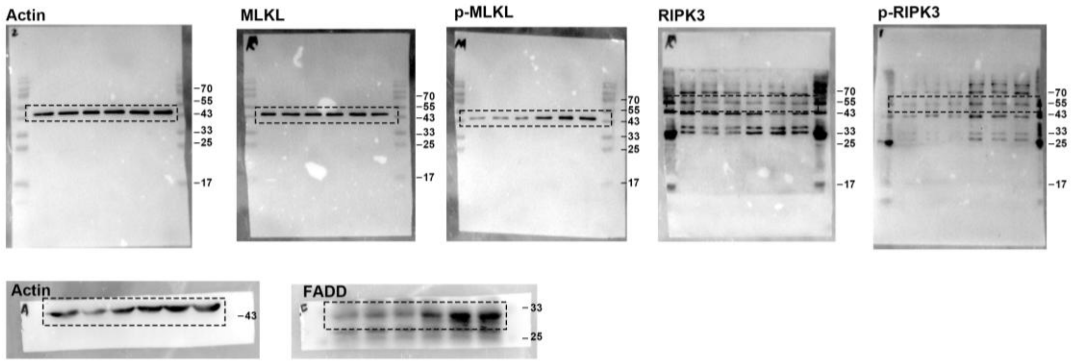

Supplementary S8 Original full length western blots for Fig 4

Fig 4C

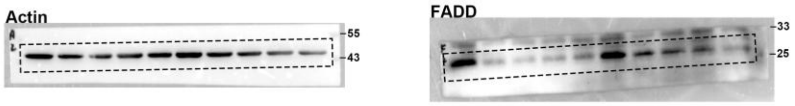

Fig 4D

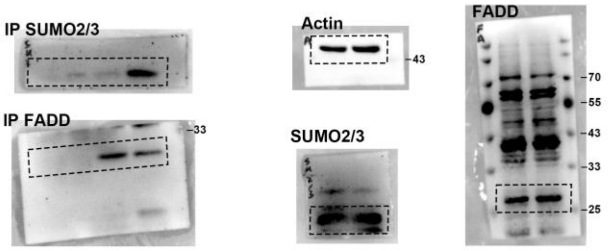

Fig 4E

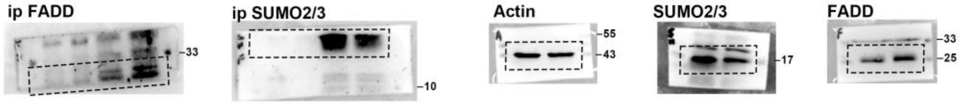

Fig 4F

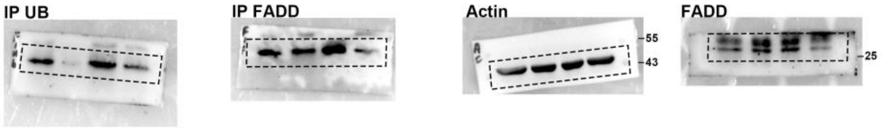

Fig 4I

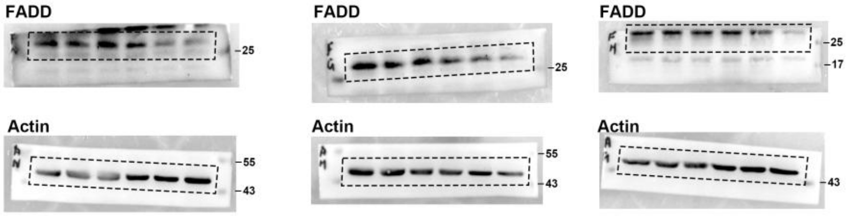

Fig 4J

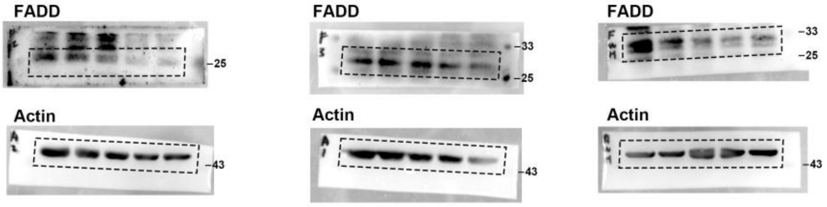

Supplementary S9 Original full length western blots for Fig 5

Fig 5A

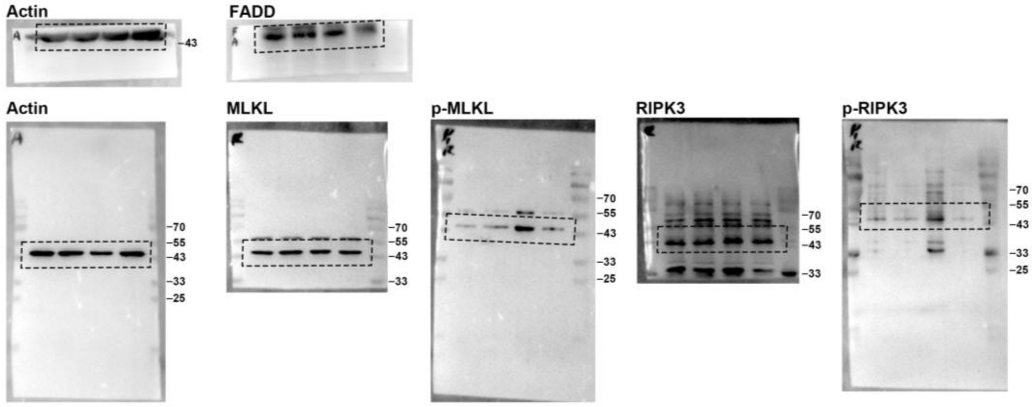

Fig 5F

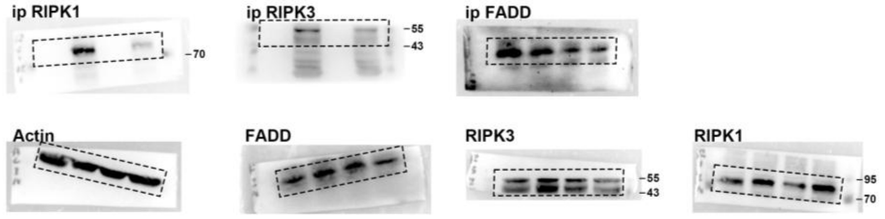

Fig 5B

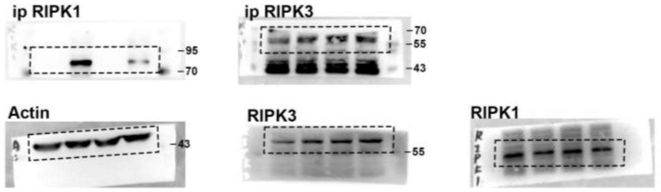

Supplementary S10 Original full length western blots for Fig 6

Fig 6H

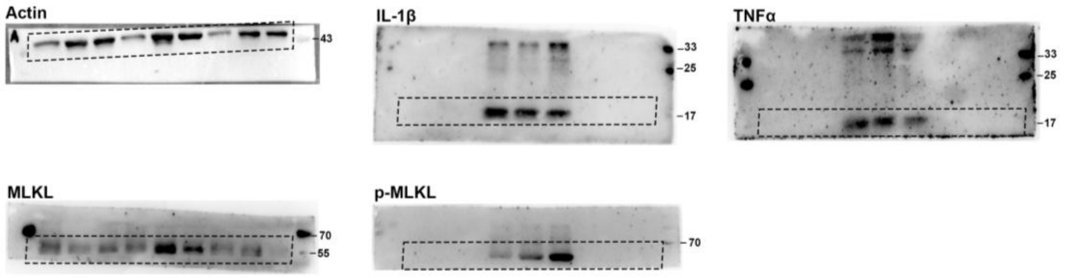

Supplementary S11 Original full length western blots for Fig S1

Fig S1E

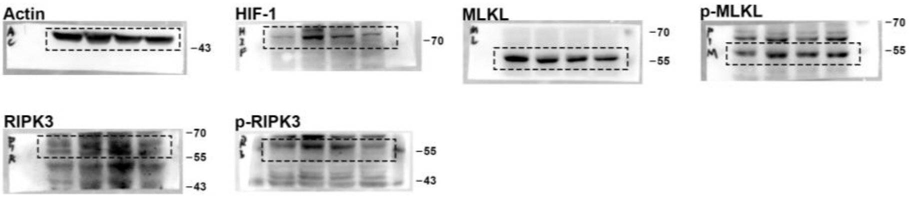

Fig S1F

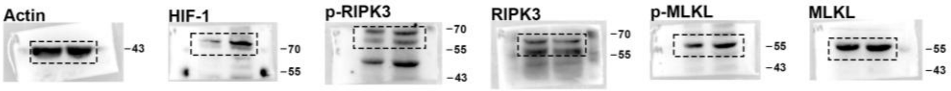

Supplementary S12 Original full length western blots for Fig S2

Fig S2A

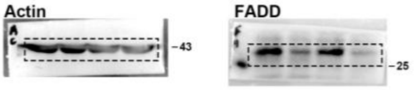

Fig S2C

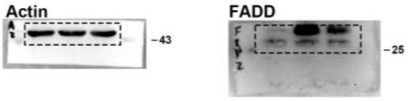

Fig S2E

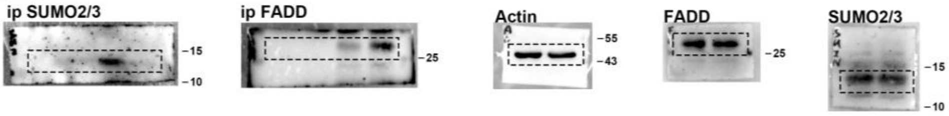

Fig S2F

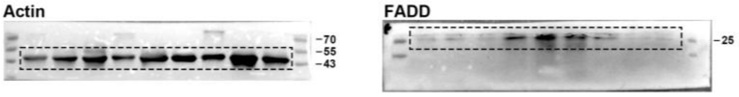

Fig S2G

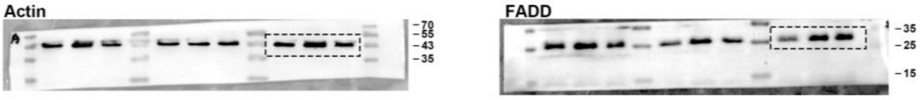

Fig S2H

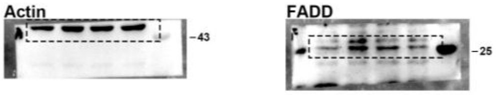

Supplementary S13 Original full length western blots for Fig S3

Fig S3A

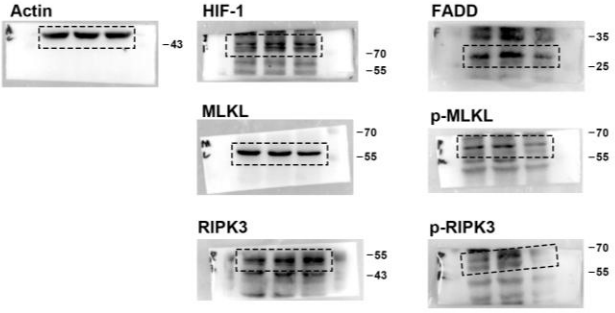

Supplement: Supplementary file 2 — Supplemental Materials [file 41419_2025_7441_MOESM2_ESM.pdf]
